# Arctigenin attenuates diabetic kidney disease through the activation of PP2A in podocytes

**DOI:** 10.1038/s41467-019-12433-w

**Published:** 2019-10-04

**Authors:** Yifei Zhong, Kyung Lee, Yueyi Deng, Yueming Ma, Yiping Chen, Xueling Li, Chengguo Wei, Shumin Yang, Tianming Wang, Nicholas J. Wong, Alecia N. Muwonge, Evren U. Azeloglu, Weijia Zhang, Bhaskar Das, John Cijiang He, Ruijie Liu

**Affiliations:** 1 Department of Nephrology, Longhua Hospital, Shanghai University of Traditional Chinese Medicine, Shanghai, China; 2 Division of Nephrology, Department of Medicine, Icahn School of Medicine at Mount Sinai, New York, NY USA; 3 Department of Pharmacology, Shanghai University of Traditional Chinese Medicine, Shanghai, 201203 China; 4 Renal Section, James J Peters Veterans Affair Medical Center, Bronx, NY USA

**Keywords:** Mechanisms of disease, Diabetic nephropathy

## Abstract

Arctigenin (ATG) is a major component of *Fructus Arctii*, a traditional herbal remedy that reduced proteinuria in diabetic patients. However, whether ATG specifically provides renoprotection in DKD is not known. Here we report that ATG administration is sufficient to attenuate proteinuria and podocyte injury in mouse models of diabetes. Transcriptomic analysis of diabetic mouse glomeruli showed that cell adhesion and inflammation are two key pathways affected by ATG treatment, and mass spectrometry analysis identified protein phosphatase 2 A (PP2A) as one of the top ATG-interacting proteins in renal cells. Enhanced PP2A activity by ATG reduces p65 NF-κB-mediated inflammatory response and high glucose-induced migration in cultured podocytes via interaction with Drebrin-1. Importantly, podocyte-specific *Pp2a* deletion in mice exacerbates DKD injury and abrogates the ATG-mediated renoprotection. Collectively, our results demonstrate a renoprotective mechanism of ATG via PP2A activation and establish PP2A as a potential target for DKD progression.

## Abstract (web summary)

Arctigenin (ATG) is the major active component of a Chinese herbal remedy known to reduce proteinuria in patients with diabetic kidney disease (DKD). Here, Zhong et al. identify PP2A as a pharmacological target of ATG in podocytes, and find that PP2A is responsible for some of the beneficial effects of ATG in mouse models of DKD.

## Introduction

Diabetic kidney disease (DKD) remains a leading cause of chronic kidney disease with limited treatment options^1^. Currently, renin–angiotensin system blockade remains a mainstay treatment that retards the DKD progression and provides partial renoprotection. However, many diabetic patients on angiotensin-converting enzyme inhibitors (ACEi) or angiotensin receptor blockades (ARB) continue to progress to end-stage renal disease, and a large unmet need remains for the development of more effective therapy for DKD patients. In China, traditional herbal medicine has been commonly used for the treatment of diabetes and its complications^2^. Among these, *Fructus Arctii* is widely used, alone or in combination with other herbal medicines, to treat diabetic patients. Notably, a small clinical observational study indicates that the use of *F. Arctii* significantly reduced the levels of proteinuria in DKD patients^3^. However, the potential mechanisms of the observed renoprotection remained obscure.

Arctigenin (ATG) is the main component of *Fructus Arctii*, which was shown to exert multiple cellular effects in vitro, including antiproliferative effects in cancer cells^4,5^ and anti-inflammatory and antioxidative effects^6–9^ in various cell types. In addition, ATG was shown to activate AMPK in vitro and in vivo^10–13^. However, the molecular mechanisms by which ATG exerts these effects remained unclear. Interestingly, recent studies reported that ATG administration in rodent models reduced renal fibrosis following unilateral ureteral obstruction^14^, which was associated with the suppression of renal inflammation and oxidative stress, and that it reduced systolic blood pressure spontaneously in hypertensive rats^15^, which was associated with reduced endothelial dysfunction and decreased NADPH oxidase-mediated superoxide anion generation. However, it was not clear whether ATG per se was responsible for the renoprotection observed in DKD patients. We now show that ATG, used as a single agent, is sufficient to significantly blunt proteinuria and attenuate the kidney disease progression in diabetic mice and that the main mechanism of action is through its binding to and activation of protein phosphatase 2 A (PP2A). PP2A, a serine/threonine phosphatase that regulates a variety of cellular processes^16^. We now show that the increased activation of PP2A by ATG results in the mitigation of NFκ-B-mediated inflammatory signaling and podocyte injury and loss in diabetic kidneys.

## Results

### ATG attenuates kidney injury in type 1 and 2 diabetic mice

**Fig. 1:**
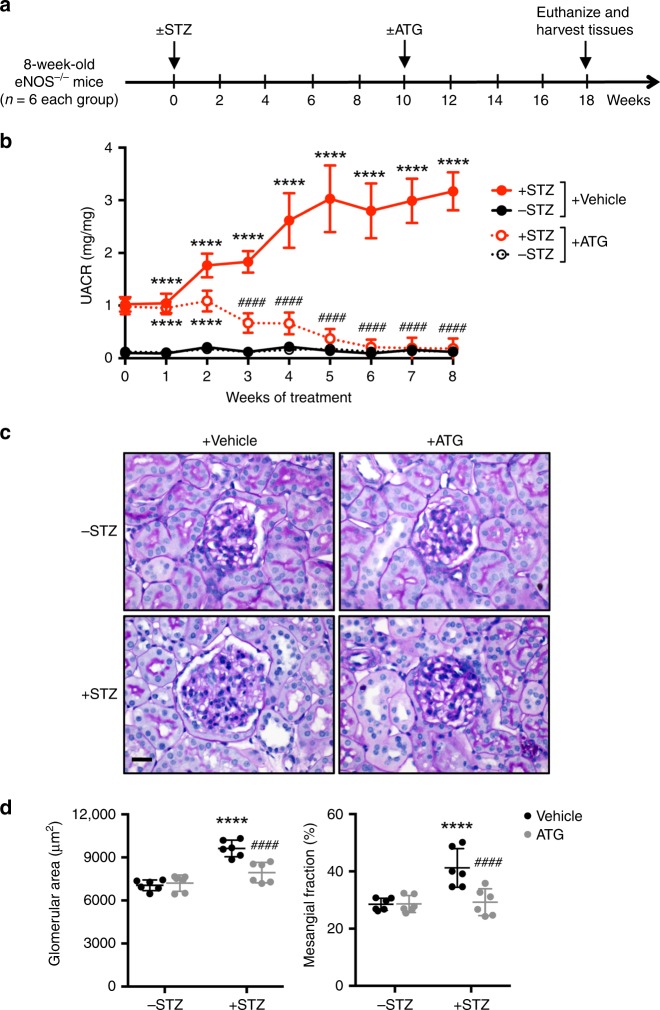
ATG treatment mitigates proteinuria and glomerular injury in diabetic eNOS^−/−^ mice. **a** Diabetes was induced in 8-week old eNOS^−/−^ mice with streptozotocin (+STZ). Vehicle-injected mice were used as nondiabetic controls (−STZ). Mice were treated with arctigenin (ATG) or vehicle by oral gavage daily at 40 mg/kg body weight for 8 weeks, starting at 10 weeks post diabetes induction. All mice were killed at 18 weeks post diabetes induction. **b** Analysis of urinary albumin-to-creatinine ratio (UACR), *n* = 6 per group. **c** Representative images of periodic acid–Schiff (PAS)-stained kidneys. Scale bar, 20 μm. **d** Quantification of the glomerular area and mesangial area fraction in diabetic and control eNOS^−/−^ mice, *n* = 6 per group. ****P < 0.0001 when compared with nondiabetic controls, ^####^*P* < 0.0001 when compared with vehicle-treated diabetic eNOS^−/−^ mice by two-way ANOVA with Tukey’s post hoc analysis. The data are represented as mean ± SD. Source data are provided as a Source Data file

**Fig. 2:**
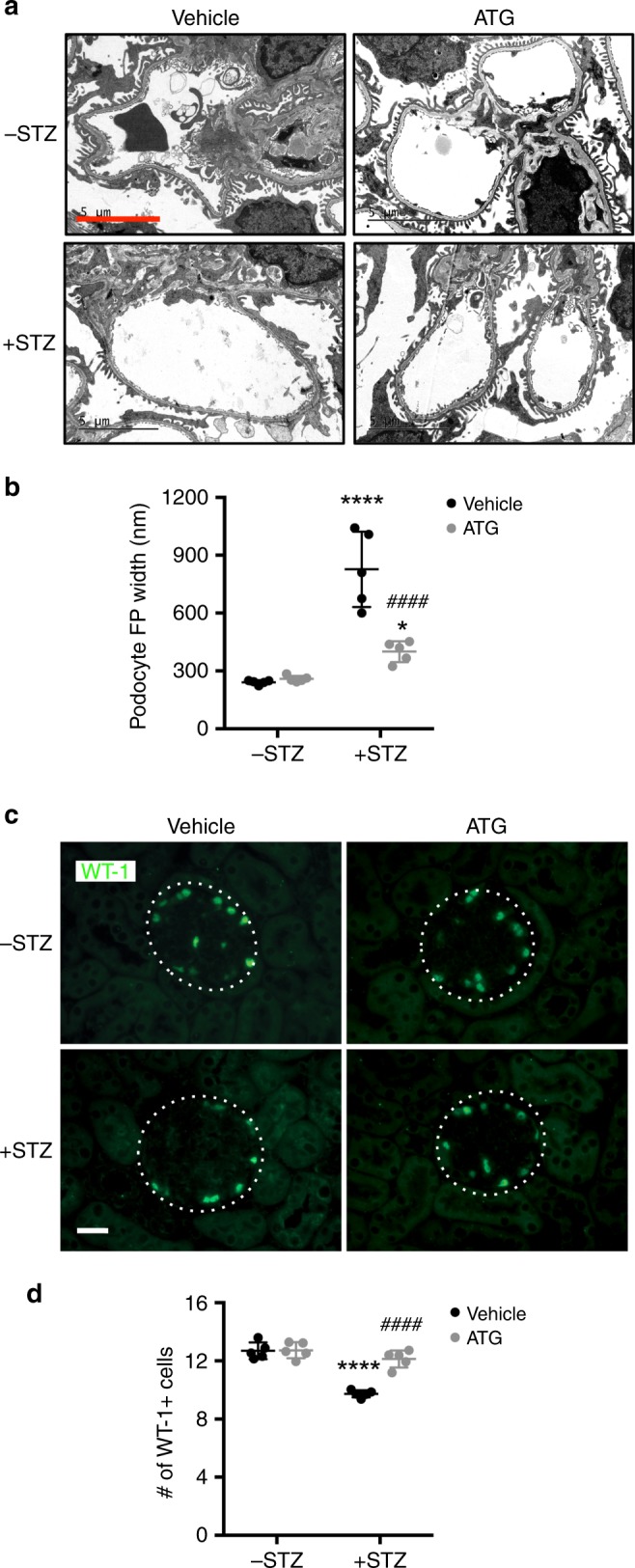
ATG treatment reduces podocyte injury and loss in diabetic eNOS^−/−^ mice. **a** Representative transmission electron microscopy images of diabetic and control eNOS^−/−^ mouse kidneys. Scale bar, 5 μm. **b** Quantification of foot process width. *P < 0.05 and ****P < 0.0001 when compared with nondiabetic controls, *n* = 5 per group. **c** Representative images of WT1 + immunofluorescence in the glomeruli of diabetic and control eNOS^−/−^ mice. Glomeruli are outlined with a white dotted circle; scale bar, 20 μm. **d** Quantification of WT1 + podocytes per glomerular cross-section, *n* = 5 per group. ****P < 0.0001 when compared with nondiabetic controls, ^####^*P* < 0.0001 when compared with vehicle-treated diabetic eNOS^−/−^ mice by two-way ANOVA with Tukey’s post hoc analysis. The data are represented as mean ± SD. Source data are provided as a Source Data file

To examine whether ATG alone can mimic the renoprotective effects of *F. Arctii* treatments in DKD, we employed two murine models of DKD. We first tested the effects of ATG on the streptozotocin (STZ)-induced experimental model of type 1 diabetes. Because the loss of endothelial nitric oxide synthase (eNOS) was shown to worsen DKD that better resembles human DKD phenotype in mice^17^, STZ was administered in eNOS-null mice (+STZ). Citrate buffer-injected eNOS^−/−^ mice served as controls (−STZ). The diabetic and control mice received either ATG (40 mg/kg of body weight) or control vehicle by oral gavage daily starting at 10 weeks after the diabetes induction when significant albuminuria was already apparent (Fig. 1a). All mice were killed after 8 weeks of ATG or vehicle treatment. As shown in the Supplementary Tables 1 and 2, the diabetic mice had increased levels of blood glucose, total cholesterol, and triglycerides and increased blood pressure as compared with the control mice, none of which were affected by the ATG treatment. The increased kidney-to-body weight ratio in the diabetic mice, however, was markedly reduced by ATG treatment (Supplementary Table 3). Notably, there was a dramatic reduction in albuminuria in ATG-treated diabetic mice, such that it was nearly abrogated by 8 weeks of the treatment (Fig. 1b). Histological analysis of periodic acid–Schiff (PAS)-stained kidneys showed that ATG treatment attenuated the glomerular hypertrophy and mesangial matrix expansion in diabetic mice (Fig. 1c, d, Supplementary Fig. 1A). Transmission electron microscopy (TEM) images showed significant podocyte foot process effacement in the diabetic mouse kidneys, which was reversed by ATG treatment (Fig. 2a, b, Supplementary Fig. 1B). Consistent with these observations, quantification of podocytes by Wilm’s tumor-1 (WT1) protein expression showed that ATG mitigated the loss of podocytes in diabetic mice (Fig. 2c, d).

We next tested the efficacy of ATG treatment in a second mouse model, *db/db*, a model of type 2 diabetes. *db/db* and nondiabetic control *db/m* mice were given either vehicle or ATG (40 mg/kg) for 6 weeks, starting 10 weeks of age when albuminuria is evident in the *db/db* mice. Consistent with the results in the STZ-induced diabetic mice, ATG treatment markedly attenuated diabetes-induced albuminuria in the *db/db* mice (Supplementary Fig. 2A–B). Glomerular injury and podocyte loss was similarly reduced in the *db/db* mice with the ATG treatment (Supplementary Figs. 2C–F). Together, these findings provide strong evidence that ATG has a potent effect on mitigating proteinuria and glomerular injury in DKD.

### ATG regulates adhesion, actin cytoskeleton, and inflammation

To elucidate the underlying mechanism of renoprotection conferred by ATG in DKD, we performed the RNA sequencing of isolated glomeruli from the diabetic and control eNOS^−/−^ mice treated with ATG or vehicle. Supplementary Fig. 3A shows the principal component analysis (PCA). The Venn diagram in Supplementary Fig. 3B shows the number of differentially expressed genes (DEGs) in the glomeruli of diabetic mice in comparison to the nondiabetic control that was reversed by ATG treatment. Supplementary Fig. 3C shows the heatmap of the top 50 DEGs in the diabetic mice that were reversed by ATG treatment, and the top 40 ATG-reversed DEGs are listed in the Supplementary Table 4. Gene enrichment analysis using the GO Biological Process, WikiPathways, and KEGG pathways showed that the regulation of cell adhesion, actin cytoskeleton, and inflammation are the major pathways enriched in ATG-reversed DEGs (Supplementary Tables 5–7). Real-time PCR on mRNAs from isolated glomeruli confirmed the changes of several key genes identified in the cell adhesion and actin regulation pathways (*Gfr, Itgb7, Washc1, Was, Zyx*) with the ATG treatment (Supplementary Fig. 3D). Indeed, ATG treatment improved adhesion and attenuated its migration of conditionally immortalized podocytes cultured in high-glucose media in comparison with normal-glucose media supplemented with high mannitol (Supplementary Fig. 4). These effects of ATG were abrogated in the presence of pan-phosphatase inhibitor okadaic acid (Supplementary Fig. 4). To better quantitate the effects of ATG on podocyte morphology, we performed a high-content image analysis of cultured podocytes as previously established^18^. It further confirmed that the ATG treatment improved podocyte morphology and adhesion under high glucose conditions (Supplementary Fig. 5). In addition, we examined the expression of two key molecules involved in the regulation of cell adhesion: focal adhesion kinase (FAK) and integrin-linked kinase (ILK). ATG treatment did not affect the expression of FAK and ILK in either normal or high glucose conditions in vitro by western blot analysis (Supplementary Fig. 6). Together with the above in vivo data, these results suggested that the potential renoprotection conferred by ATG treatment is in part through improved podocyte adhesion, decreased motility, and the regulation of actin cytoskeleton, resulting in increased podocyte viability and function in the diabetic kidneys.

### PP2A is a direct target protein of ATG in kidney cells

**Fig. 3:**
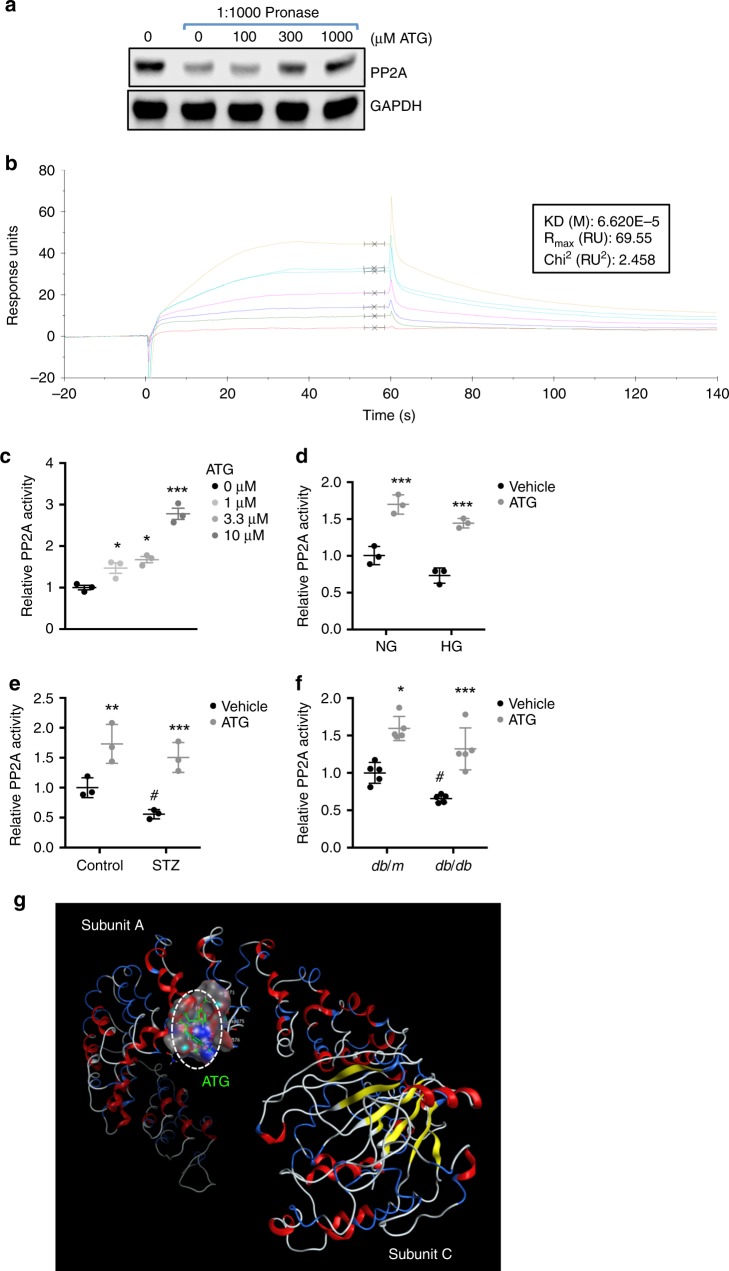
ATG binds to PP2A to enhance its activity. Drug Affinity Responsive Target Stability (DARTS) assay was performed to test the direct binding of ATG to PP2A in renal cells. **a** HEK293T cell lysates were pre-incubated with various concentrations of ATG as indicated at 25 °C for 1 h prior to digestion with pronase (0 or 1:1000 dilution) for 20 min. Lysates were then probed for PP2A expression, with GAPDH as a loading control. **b** Surface Plasmon Resonance (SPR) assay with Biacore showing the steady-state fit of binding between ATG and PP2A. **c** PP2A activity in HEK293T cells treated with various ATG concentrations as indicated for 2 h. Total PP2A activity is expressed as a relative fold change to no ATG treatment. *n* = 3 experiments. *P < 0.05 and ***P < 0.001 when compared with control by one-way ANOVA with Tukey’s post hoc analysis. **d** PP2A activity in conditionally immortalized human podocytes exposed to normal glucose and high mannitol (NG; 5 mM glucose and 25 mM mannitol) or high glucose (HG; 30 mM glucose) conditions for 24 h prior to treatment with ATG (10 μM) or vehicle for 2 h. Total PP2A activity is expressed as a relative fold change to NG vehicle control. ***P < 0.001 compared wqith vehicle control; *n* = 3. **e** PP2A activity in isolated glomeruli of control and diabetic eNOS^−/−^ mice. Total PP2A activity is expressed as a relative fold change to vehicle-treated nondiabetic control. *n* = 3 per group. **P < 0.01 and ***P < 0.001 compared with vehicle control; ^#^*P* < 0.05 compared wqith vehicle-treated control mice. **f** PP2A activity in isolated glomeruli of *db/m* and *db/db* mice. Total PP2A activity is expressed as a relative fold change to vehicle-treated *db/m* mice. *n* = 5 per group. *P < 0.05 and ***P < 0.001 compared with respective vehicle control; ^#^*P* < 0.05 compared with vehicle-treated *db/m* mice by two-way ANOVA with Tukey’s post hoc analysis. *P < 0.05 and ***P < 0.001 when compared with control by two-way ANOVA with Tukey’s post hoc analysis. The data are represented as mean ± SD. **g** Computational prediction of ATG binding to PP2A crystal structure. Docking values for ATG for the binding using the virtual screening: Value S = −8.7414; E configuratio*n* = 3.2185; E place = -51.5684; E score1 = −8.7414. Source data are provided as a Source Data file

To determine the molecular mechanisms of ATG’s action on podocyte function, we next utilized the drug affinity responsive target stability (DARTS) method^19–21^ to identify potential ATG-binding proteins in cultured renal cells. DARTS assay is based on the principle that the binding of a small molecule compound to the target protein changes the protein conformation, leading to increased protein stability and protection against proteolysis. To analyze the ATG-bound proteins, mass spectrometry analysis was performed following the DARTS assay with pronase in HEK293T cells treated with vehicle or ATG (*n* = 1 sample per experimental condition). Supplementary Table 8 shows the list of the top 8 of the ATG-bound proteins. As the above data were generated from one sample per experimental condition, we validated these findings by DARTS assay followed by western blot analysis. The top two ATG-bound proteins were tubulin beta chains, but we were not able to validate their interactions with ATG by DARTS/western blot analysis, using two different tubulin antibodies recognizing different epitopes (Supplementary Fig. 7). The third protein on the list was the catalytic subunit beta of PP2A. PP2A is a ubiquitously expressed serine/threonine phosphatase that exists in cells predominantly as a core dimer, consisting of catalytic subunit C and structural subunit A, and a regulatory subunit B^22^. DARTS/western blot analysis indeed confirmed the interaction between PP2A and ATG in HEK293T cells in a dose-dependent manner (Fig. 3a).

Interestingly, the computational modeling analysis of ATG binding to PP2A using the known structure of PP2A core dimer (Protein Data Bank, 2IAE) predicted its binding to the subunit A, near the junction of A and C subunits (Fig. 3g, Supplementary Fig. 8A). The modeling further showed that this putative binding would be similar to those of two PP2A agonists, fingolimod and forskolin as suggested by other studies^23^ (Supplementary Fig. 8B–C), but different from that of PP2A inhibitor okadaic acid (Supplementary Fig. 8D)^23^. In addition, surface plasmon resonance (SPR) assay demonstrated the direct binding between ATG and PP2A subunit A, with equilibrium dissociation constant (*K*_*D*_) of 6.6 × 10^−5^ m for steady-state fit (Fig. 3b, Supplementary Fig. 9) Interestingly, the quality of fits is improved for a two-state model with a *K*_*D*_ of 3.3 × 10^-7^ M. This may suggest a conformational change occurred after binding of ATG with PP2A (Supplementary Fig. 9). We also tested the binding for forskolin and fingolimod (FTY720), two known agonists of PP2A. Interestingly, as shown in Supplementary Fig. 10, forskolin binds with a similar KD with PP2A (*K*_*D*_ of 8.0 × 10^-5^ M for steady-state fit and *K*_*D*_ of 4.8 × 10^-9^ for two-state fit). However, the stoichiometry for fingolimod-PP2A binding is more complex with a poor fit (Supplementary Fig. 11). In addition, the steady-state binding was not completely reached for forskolin and FTY. Together these results confirm that PP2A is a direct target of ATG.

Moreover, ATG treatment resulted in increased PP2A activity in a dose-dependent manner in HEK293T cells (Fig. 3c). ATG treatment also increased PP2A activity in podocytes cultured under normal or high glucose conditions in vitro (Fig. 3d). We further confirmed that ATG treatment increased the PP2A activity in the glomeruli of diabetic eNOS^−/−^ and *db/db* mice in vivo (Fig. 3e, f). ATG treatment did not affect the expression level of PP2A in cultured podocytes in vitro or in glomeruli of diabetic mice in vivo (Supplementary Fig. 12), indicating that the binding of ATG to PP2A increases its activity.

### ATG inhibits NF-κB pathway via PP2A activation in podocytes

**Fig. 4:**
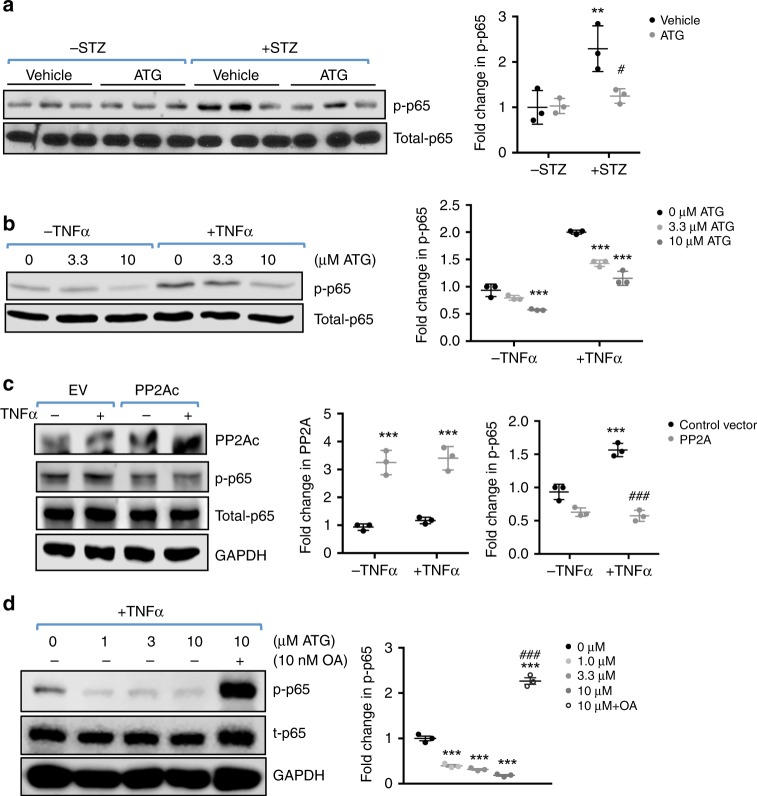
ATG reduces TNF-α-induced inflammation through PP2A activation. **a** Levels of p65 NF-κB phosphorylation (p-p65) in the kidney glomeruli of control and diabetic mice with or without ATG treatment. Quantification of p-p65 normalized to total p65 (t-p65) is shown on the right. *n* = 3 per group. **P < 0.01 vs. nondiabetic mice, ^#^*P* < 0.05 vs. vehicle-treated diabetic mice by two-way ANOVA with Tukey’s post hoc analysis. **b** Levels p-p65 in cultured podocytes treated with or without TNF-α. Quantification of p-p65 normalized to total p65 is shown on the right, *n* = 3. ***P < 0.001 vs. 0 μM ATG. **c** Overexpression of PP2A catalytic subunit (PP2Ac) by transient transfection reduces p65 NF-κB activation in cultured podocytes in vitro. EV, empty vector-transfected. Quantification of PP2A levels normalized to GAPDH and p-p65 normalized to t-p65 is shown on the right. ***P < 0.001 vs. EV; ^###^*P* < 0.001 vs. TNF-α-treated EV. **d** Levels p-p65 in cultured podocytes treated with ATG in presence or absence of okadaic acid (OA). Quantification of p-p65 levels normalized to GAPDH is shown on the right, *n* = 3. ***P < 0.001 vs. 0 μM ATG; ^###^*P* < 0.001 vs. TNF-α-treated EV by 1-way ANOVA with Tukey’s post hoc analysis. The data are represented as mean ± SD. Source data are provided as a Source Data file

One of the key features of DKD is increased inflammation^24^. PP2A is shown to be a potent negative regulator of multiple inflammatory signaling pathways^25,26^, including the dephosphorylation and repression of p65 NF-κB^27,28^. Therefore, we next examined whether ATG may dampen the NF-κB-mediated renal inflammation by enhancing PP2A activity in DKD. Indeed, western blot analysis showed that the increased p65 NF-κB phosphorylation in diabetic eNOS^−/−^ mouse glomeruli was markedly reduced with ATG treatment (Fig. 4a). Using cultured podocytes, we further confirmed that ATG treatment inhibited the activation of TNF-α-mediated p65 activation (Fig. 4b) and that the increased PP2A expression by transient transfection mimicked this inhibition (Fig. 4c). Conversely, the inhibition of PP2A activity by okadaic acid abolished the effects of ATG on p65 phosphorylation in cultured podocytes (Fig. 4d). Together, these data indicate that the renoprotection conferred by ATG is in part mediated through its antiinflammatory effects of PP2A in the diabetic kidneys.

### PP2A regulates podocyte actin cytoskeleton through DBN1

**Fig. 5:**
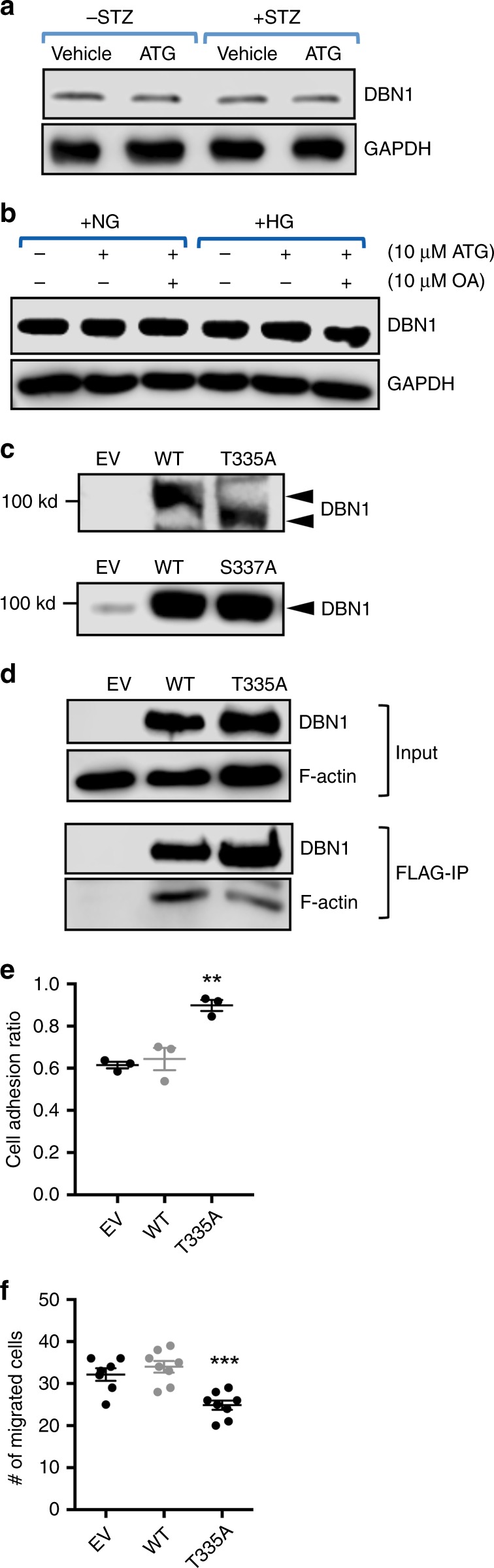
PP2A regulates podocyte actin cytoskeleton and motility through DBN1 regulation. **a** Western blot of DBN1 in diabetic and control eNOS^−/−^ mouse glomeruli treated with vehicle or ATG. **b** Western blot of DBN in podocytes cultured under normal or high glucose conditions with ATG and/or okadaic acid (OA) treatments as indicated. **c** Phos-tag gel and western blot analysis of immortalized podocytes stably transfected with either empty vector (EV) or expression vector expressing either wildtype (WT) DBN1, DBN1 T335A (top), or DBN1 S337A (bottom). **d** Lysates of podocytes stably expressing FLAG-tagged DBN1 (WT or T335A) were immunoprecipitated with anti-FLAG antibody and immunoblotted for F-actin. The top panels show the western blot total input and the bottom panel shows the immunoprecipitated proteins. **e** Adhesion assay in podocytes stably expressing DBN1 T335A in comparison with those with empty vector (EV) or DBN1 WT expression, *n* = 3. **f** Migration assay of podocytes stably expressing DBN1 T335A in comparison with those with empty vector (EV) or DBN1 WT expression, *n* = 7. **P < 0.01 or ***P < 0.001 compared to all other groups by one-way ANOVA with Tukey’s post hoc analysis. The data are represented as mean ± SD. Source data are provided as a Source Data file

To further dissect how ATG might improve podocyte function in the diabetic mice, we next performed a mass spectrometry analysis of PP2A-interacting proteins that were immunoprecipitated from cultured podocytes with FLAG-tagged PP2A overexpression. Supplementary Table 9 shows the top 20 proteins that were identified from this screen (*n* = 1 sample per experimental condition), many of which were related to actin cytoskeleton and inflammation. Among these, two of the actin cytoskeleton-related proteins, CKAP4 and Drebrin-1 (DBN1), were highly expressed in the glomeruli as compared to tubules, and their mRNA expression was modestly reduced in the glomeruli of DKD patients according to Nephroseq data sets (*nephroseq.org*) (Supplementary Fig. 13). We chose to focus on DBN1 for further analysis, as its expression was previously shown to localize to the podocyte foot processes^29^. In addition, F-actin is known to mediate podocyte foot process formation^30^, and DBN1 was shown to interact with F-actin to stabilize actin filaments and regulate cell migration^31,32^. However, we could not confirm whether the protein levels of DBN1 were indeed reduced in the human diabetic kidneys since currently available antibodies are not optimal for immunohistochemical staining. Western blot analysis of isolated mouse glomeruli, however, showed that there were no alterations in the DBN1 levels in the diabetic mice (Fig. 5a) or cultured podocytes under high-glucose conditions (Fig. 5b), consistent with the mRNA data of isolated glomeruli from other murine models of diabetes (*nephroseq.org*). Thus, whether glomerular DBN1 expression is indeed decreased in the late stages of DKD (as in human diabetic kidneys) rather than in early stages (as in mouse diabetic kidneys) remains to be explored. Similarly, ATG treatment had no effects on DBN1 expression in the isolated glomeruli of control and diabetic mice or in cultured podocytes in vitro (Fig. 5a, b).

Among its various putative phosphorylation sites on DBN1, S142 was previously reported to be critical for its interaction with F-actin^31^. Therefore, we determined the potential phosphorylation sites of DBN1 that are affected by ATG/PP2A by immunoprecipitation/mass spectrometry analysis in the vehicle- and ATG-treated podocytes, and in podocytes with shRNA-mediated PP2A knockdown. The results indicated that although S142 was indeed phosphorylated in cultured podocytes (Supplementary Table 10), it was not significantly affected by ATG nor by PP2A knockdown. However, phosphorylation of T335/S337 on DBN1 was reduced by ATG treatment and increased with PP2A knockdown (Supplementary Table 10). Although the mass spectrometry analysis indicated single phosphorylation of the target peptide (FDR < 0.01), it could not whether T335 or S337was phosphorylated. Therefore, to ascertain the site of phosphorylation, FLAG-tagged DBN1 wildtype or with mutation of either T335 or S337 to alanine (T335A or S337A) was introduced in HEK293T cells. Phos-tag gel analysis that detects the mobility shift of phosphorylated proteins^33^ indicated that T335 is indeed the site of phosphorylation and not S337, as only the T335A mutation affected its migration in comparison to DBN1 WT (Fig. 5c). Importantly, less F-actin was pulled down with DBN1 by anti-FLAG immunoprecipitation in cells expressing DBN1 T335A in comparison with those expressing the DBN1 WT (Fig. 5d), suggesting that the regulation of T335 phosphorylation is also important for interaction with F-actin. We next examined the effects of DBN1 in podocyte adhesion and migration by transient transfection. In comparison to the control vector- or DBN1 WT1 vector-transfected podocytes, DBN T335A-transfected cells showed increased adhesion and reduced the migration (Fig. 5e, f), suggesting that T335 phosphorylation of DBN1 induces podocyte migration and detachment by interacting with F-actin. Taken together, these results suggest that PP2A activation through ATG improves podocyte adhesion in part through the dephosphorylation of DBN1 T335, uncovering another important mechanism of podocyte cytoskeletal regulation.

### Podocyte-specific knockout of PP2A aggravates DKD in mice

**Fig. 6:**
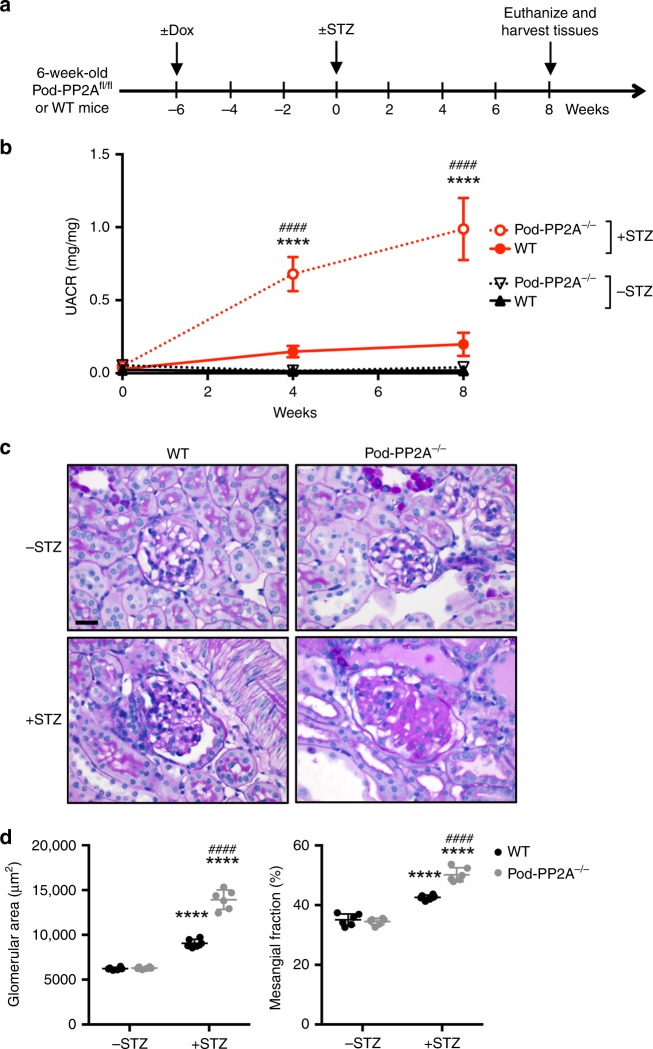
Podocyte-specific loss of PP2A worsens DKD. **a** Schematics of the experimental design. Doxycycline (Dox) was administered in 6-week old wildtype (WT) or Pod-PP2A^−/−^ mice. Diabetes was induced with STZ at 6 weeks post Dox induction. All mice were killed 8 weeks after diabetes induction, *n* = 6 mice per group. **b** Analysis of urinary albumin-to-creatinine ratio (UACR). ***P < 0.001 and ****P < 0.0001 when compared with WT diabetic mice; *n* = 6. **c** Representative PAS images. Scale bar, 20 μm. **d** Quantification of the glomerular area and mesangial matrix fraction in diabetic and control mice, *n* = 6. ****P < 0.0001 vs. respective nondiabetic mice; ^####^*P* < 0.0001 vs. WT diabetic mice by two-way ANOVA with Tukey’s post hoc analysis. The data are represented as mean ± SD. Source data are provided as a Source Data file

**Fig. 7:**
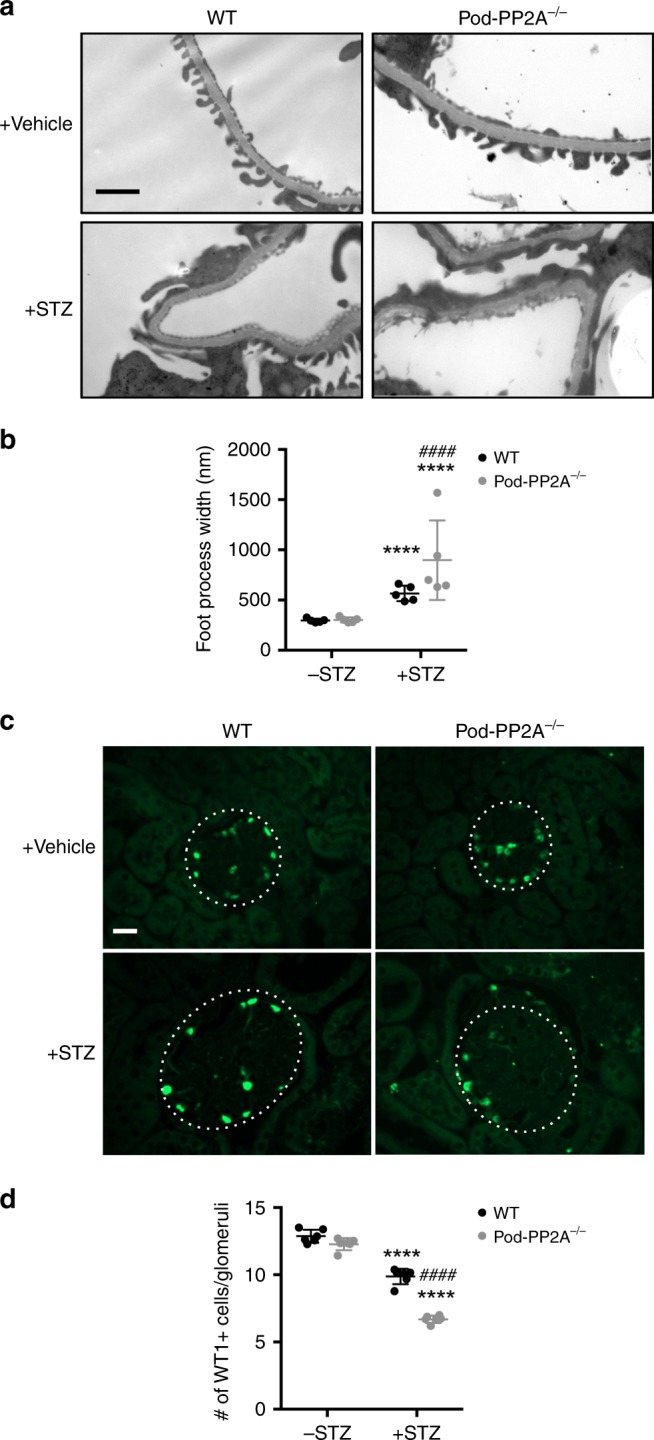
Podocyte-specific loss of PP2A increases podocyte injury and loss. **a** Representative transmission electron microscopy images of kidneys of diabetic and control mice. Scale bar, 1 μm. **b** Quantification of foot process width. *n* = 5. **c** Representative immunofluorescence images of WT1 + podocytes in glomeruli of diabetic and control mice. Glomeruli are outlined with a white dotted circle; scale bar, 20 μm. **d** Quantification of WT1 + podocytes per glomerular cross-section. *n* = 5 per group. ****P < 0.0001 vs. respective nondiabetic mice, ^####^*P* < 0.0001 vs. WT diabetic mice by two-way ANOVA with Tukey’s post hoc analysis. The data are represented as mean ± SD. Source data are provided as a Source Data file

To confirm the in vivo role of PP2A as a key phosphatase protecting the podocytes from diabetes-induced injury, we generated inducible podocyte-specific PP2A knockout mice by crossing the conditional PP2A-null mice (*Ppp2r1a*^*fl/fl*^)^34^ with Nphs2-rtTA and tetO-Cre transgenic mice. Podocyte-specific PP2A loss in *Ppp2r1a*^fl/fl^;Nphs2-rtTA;tetO-Cre mice was induced with 0.5 mg/ml doxycycline (Dox) in 5% sucrose-supplemented drinking water starting at 6 weeks of age (hereafter referred to as Pod-PP2A^−/−^ mice; Fig. 6a). Dox-treated control littermates (without either Nphs2-rtTA or tetO-Cre transgenes) served as wildtype controls (WT). Diabetes was induced in 12-week old WT and Pod-PP2A^−/−^ mice by STZ injection ( +STZ) (Fig. 6a). Mice that received sodium citrate buffer vehicle (−STZ) served as nondiabetic controls. All mice were killed at 8 weeks after STZ or vehicle injection. Pod-PP2A^−/−^ mice developed mild albuminuria (twofold to threefold increase) without any remarkable histologic changes at 4 months of age (Fig. 6b). At 8 weeks post diabetes induction, the extent of hyperglycemia in the diabetic Pod-PP2A^−/−^ or WT mice was similar, but the diabetes-induced renal hypertrophy was more pronounced in the in diabetic Pod-PP2A^−/−^ mice (Supplementary Table 11). Moreover, diabetic Pod-PP2A^−/−^ mice displayed significantly worsened albuminuria, glomerular hypertrophy, and mesangial expansion as compared with the diabetic WT mice (Fig. 6b–d, Supplementary Fig. 14). These changes were associated with increased podocyte foot process effacement and loss in comparison with the diabetic WT mice (Fig. 7a–d). These results demonstrate that the podocyte-specific loss of PP2A leads to aggravated diabetic glomerulopathy and accelerated DKD progression.

### Effect of ATG is abrogated in diabetic Pod-PP2A^−/−^ mice

**Fig. 8:**
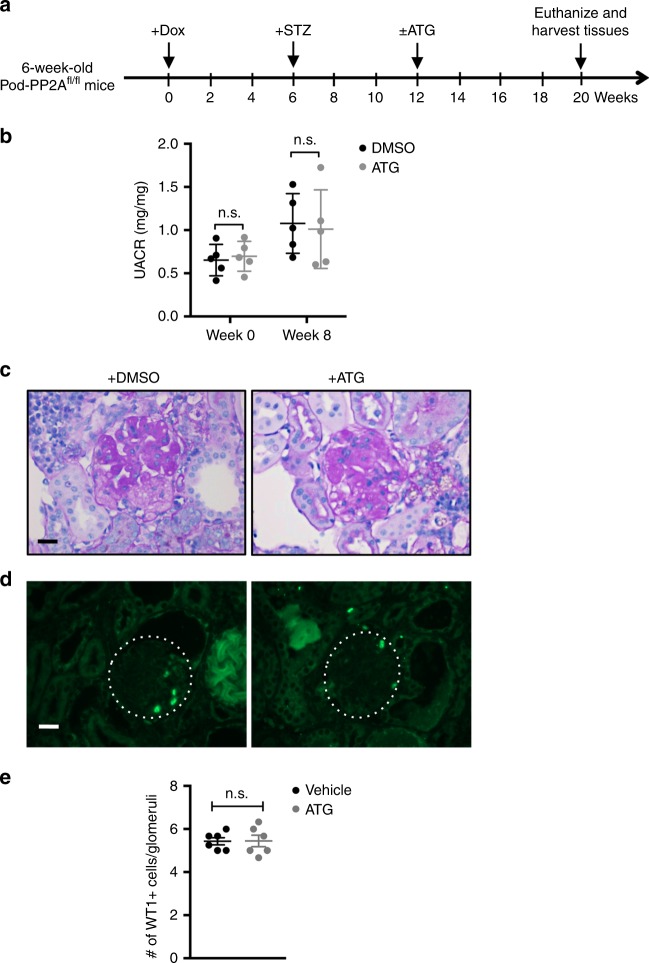
Renoprotection by ATG is abrogated in diabetic Pod-PP2A^−/−^ mice. **a** Schematics of the experimental design. Doxycycline (Dox) was administered in 6-week old Pod-PP2A^−/−^ mice. Diabetes was induced with STZ at 6 weeks postDox induction. ATG or DMSO vehicle was administered daily starting at 6 weeks post diabetes induction for 8 weeks. All mice were killed 14 weeks after diabetes induction. **b** Analysis of urinary albumin-to-creatinine ratio (UACR) at week 0 or week 8 of ATG treatment, which were not significant (n.s.) between treatment groups by two-way ANOVA with Tukey’s post hoc analysis. *n* = 5. **c** Representative PAS images. Scale bar, 20 μm. **d**, **e** Representative immunofluorescence images **d** and quantification of WT1^+^ cells **e** are shown per glomerular cross-section. The data are represented as mean ± SD. Two-tailed *t* test was performed to compare the data between two groups. Source data are provided as a Source Data file

To further confirm whether the renoprotective effects of ATG were indeed mediated by PP2A, we treated another set of diabetic Pod-PP2A^−/−^ mice with either ATG or vehicle for 8 weeks starting at 6 weeks post STZ injection (Fig. 8a). As anticipated, ATG treatment was ineffective against diabetes-induced albuminuria, glomerular injury, and podocyte loss (Fig. 8b–e), clearly demonstrating that the renoprotection conferred by ATG is lost in the absence of PP2A activity in podocytes. Taken together, these results demonstrate that ATG acts primarily through the regulation of PP2A activity, and uncovers a role of PP2A as a key regulator of diabetes-induced podocyte injury and DKD progression.

## Discussion

Chinese traditional herbal medicines have been widely used to treat CKD including DKD with promising outcomes^35,36^. However, the active components and the underlining mechanisms of action remain unclear. In this study, we demonstrated that ATG is a natural agonist of PP2A that has potent renoprotective effects in both type 1 and type 2 diabetic animal models. Our data demonstrate that increased activation of PP2A by ATG results in dampening of NF-κB-mediated inflammatory effects and increased stability of podocyte actin cytoskeleton through DBN1 dephosphorylation (summarized in the Supplementary Fig. 15). To our knowledge, this is the first study to demonstrate the detailed cellular and molecular mechanisms of a compound from natural herbal medicines in providing protection in kidney disease.

Transcriptomic profiling of isolated glomeruli from ATG-treated diabetic mice showed that ATG confers renoprotection through the regulation of inflammation and adhesion/actin cytoskeleton pathways of glomerular cells. This is consistent with the observation that renal inflammation is a key component of DKD^37^ and that increased podocyte motility and detachment are considered to be major mechanisms of podocyte loss in the diabetic kidneys^38^. Our data indicate that the ATG treatment reduced NF-κB phosphorylation through the activation of PP2A. In line with these observations, other studies have shown that PP2A inhibits NF-κB activation through its interaction with p65^27,39^ and inhibition of IKKα^40^ or IKKβ^41^. It has been also shown that NF-κB activation can be regulated by PP2A through inhibition of AKT and subsequent inhibition of IKK^42^. In addition, the antiinflammatory effect of PP2A could be through inhibition of MAPK^26^. Interestingly, it has been shown that ATG could interact with MKK-1 and adiponectin receptor 1^43,44^. However, these target molecules were not in the list of proteins that we identified from our DARTS-Mass spectrometry experiments with ATG. The role of these proteins in DKD remains to be determined.

To better understand the downstream effects of ATG-induced PP2A regulation in renal cells, we performed a mass spectrometry analysis of PP2A-interacting proteins. Consistent with above, several top-ranked proteins are related to inflammation and actin cytoskeleton. Among the PP2A-interacting proteins, we were particularly interested in DBN1, which was previously shown to localize in podocyte foot process^29^. DBN1 is an actin-associating protein enriched at the cell-cell junction, forming a specific microfilament anchorage system in polarized epithelial cells^45^. In neuronal cells, DBN1 couples dynamic microtubules to F-actin in growth cone filopodia and organizes F-actin in dendritic spines, having a critical role in neuritogenesis and synaptic plasticity^46^. The interaction between DBN1 and F-actin was shown to be regulated by Cdk5-mediated phosphorylation of S142 located in the coiled-coil domain of DBN1^31^. Our data indicate that the phosphorylation of DBN1 at T335, rather than S142, was reduced by ATG treatment in podocytes through the activation of PP2A, and to be critical for its interaction with F-actin and actin cytoskeleton regulation in podocytes. Thus, our results demonstrate a previously unidentified mechanism of actin cytoskeleton regulation in podocytes. Interestingly, the glomerular expression of DBN1 mRNA was modestly reduced in DKD patients according to Nephroseq data sets. However, this was not observed in the experimental mouse models in the same database. Consistent with this, we also did not detect a change in its protein expression in the glomeruli of our diabetic mouse model. However, the potential discrepancy in gene expression between mouse and human diabetic kidneys is not surprising, given that the human diabetic kidney biopsies are mostly from late stages of DKD, whereas mouse diabetic mice develop only early DKD phenotypes, and as demonstrated by previous comparative transcriptome analyses^47^. It is also plausible that the reduction of DBN1 in human diabetic glomeruli is attributed by the loss of podocytes in advanced DKD, rather than in the reduction of DBN1 gene per se.

The role of PP2A in kidney disease such as DKD has never been shown previously in vivo. One study suggests that PP2A is a negative regulator of TGF-β1-induced TAK1 activation in cultured mesangial cells^48^. To further confirm the role of PP2A in podocyte injury in DKD, we generated an inducible podocyte-specific PP2A knockout mouse model. Using this model, we confirmed that knockout of PP2A in podocytes aggravated podocyte injury and DKD, confirming a critical role of PP2A in vivo in DKD. Our data suggest that PP2A could be a potential drug target for the treatment of DKD through the improvement of podocyte injury.

In addition to the PP2A-mediated mechanisms that we identified above, ATG was previously shown to be an activator of AMPK to improve diabetes^11^. However, in our studies, treatment of ATG did not affect hyperglycemia in these mice. Activation of AMPK pathway has been shown to have renoprotective effects in DKD^11^. Therefore, ATG may protect against DKD injury through multiple pathways. In addition, ATG has been shown to have anticancer effects^5^. PP2A is a tumor suppressor protein that is genetically altered or functionally inactivated in many cancers^16^, highlighting a need for its therapeutic reactivation. Our data suggest that ATG, as a natural agonist of PP2A, may have tumor-suppressive effects through activation of PP2A. ATG and its analogs could be developed as potential cancer therapy. Therefore, we believe that our findings may have broader implications than in kidney disease alone.

Our study demonstrates that PP2A activity is reduced in the diabetic glomeruli while the total protein levels did not change. Interestingly, it has been shown that PP2A activity is increased in several other tissues such as livers and muscles in diabetic condition^49^ and may contribute to endothelial cell injury^50^. We have not addressed the role of PP2A in endothelial cells in the current study. Further studies are required to determine the role of PP2A in other kidney cells including glomerular endothelial cells and tubular cells. We are aware that the *K*_D_ of ATG binding with PP2A was relatively low. However, this range of *K*_D_ is not uncommon for small molecules bindings with proteins^51,52^. On the other hand, these data suggest that we could further modify the structure of ATG to improve the binding affinity with PP2A and we might be able to get more potent PP2A agonists than ATG or forskolin. Another limitation of the study is that we have not addressed the interaction between PP2A and tubulin, as this has been reported previously in several studies^53–56^. Therefore, although we did not find a direct interaction between PP2A and tubulin by our assays using two different antibodies against tubulin beta chains, PP2A may yet interact with tubulin in other renal cell types and their interaction cannot be fully ruled out. In this study, we chose to focus on the role of DBN1 in the regulation of F-actin in podocytes, because actin is involved directly in the regulation of podocyte foot process while tubulin is involved more in the regulation of major processes of podocytes^30^. As a potential drug target to treat DKD, it would also be important to determine whether the chronic activation of PP2A may have any detrimental effects in various organs.

In conclusion, we demonstrate here ATG and other analogs that increase PP2A activity may be developed as a potential treatment for DKD. Further exploration of ATG analogs and others based on the interaction between ATG and PP2A may help to identify more potent and safe drugs to move to clinical trials for patients with DKD.

## Methods

### Mouse models

Male eNOS homozygous knockout (eNOS^−/−^) mice on a C57BL/6 J background (stock# 002684) and *db/db* mice in C57BLKS/J background (stock # 000642) were purchased from The Jackson Laboratory. For eNOS^−/−^ mice, diabetes was induced in 8-week old mice with intraperitoneal administration of STZ (Sigma #S0130, dissolved in 0.1 m citrate buffer, PH 4.5) at 50 mg/kg after 4–6 h of food deprivation each day for 5 consecutive days. Citrate buffer-injected mice served as nondiabetic controls. Ten weeks after diabetes induction, mice were given ATG (Cayman Chemicals, #14913) dissolved in 5% DMSO by oral gavage at a dose of 40 mg/kg body weight/day for 8 weeks. In total, 5% DMSO vehicle-treated mice served as controls. For *db/db* mice, 10-week old mice were treated with either vehicle or ATG by oral gavage at a dose of 40 mg/kg body weight/day for 6 weeks. Blood glucose, urinary albumin, and body weight were monitored weekly. Inducible podocyte-specific PP2A knockout mice were generated by crossing PP2A floxed mice (PP2A regulatory subunit A alpha, *Ppp2r1a*) with NPHS2-rtTA and tetO-Cre transgenic mice; all three mouse lines were in the FVB/NJ background and obtained from The Jackson Laboratory (stock # 017441, 008202, and 008244). Loss of podocyte-specific PP2A in *Ppp2r1a*^fl/fl^;NPHS2-rtTA;tetO-Cre was induced with 0.5 mg/ml doxycycline in drinking water supplemented with 5% sucrose for 1 week. Diabetes was induced with STZ as above. All procedures performed in the study involving animals were in accordance with the ethical standards of and protocol (#15-1402) approved by the Institutional Animal Care and Use Committee at the Icahn School of Medicine at Mount Sinai, New York, NY.

### Blood pressure monitoring

Blood pressure was measured using the CODA programmable non-invasive tail-cuff sphygmomanometer (Kent Scientific, Torrington, CT) on conscious mice. Mice were initially subjected to an acclimation period of five cycles prior to blood pressure assessment. Subsequently, systolic blood pressure (SBP) was measured in each mouse for 60 continuous cycles and an average of SBP was quantified.

### Urine albumin and creatinine measurement

Urine albumin was measured using an ELISA kit (Bethyl Laboratory, Houston, TX), and urine creatinine was measured using a colorimetric assay kit (Cayman, Ann Arbor, MI.)

### Kidney histology

Kidneys were removed and fixed with 4% paraformaldehyde 16 h at 4 °C. The 4-μm sections were cut from paraffin-embedded kidney tissues. Sections were stained with PAS) for histology analysis. Assessment of the mesangial and glomerular cross-sectional areas was performed by pixel counts on the kidney section in a blinded fashion, under × 400 magnification (Zeiss AX10 microscope, Carl Zeiss Canada Ltd, Toronto, ON, Canada) as previously described^32,33^. In brief, digitized images were scanned and profile areas were traced using ImageJ. The mean glomerular tuft volume was determined from the mean glomerular cross-sectional area by light microscopy. The glomerular cross-sectional area was calculated based on the average area of 30 glomeruli in each group, and the glomerular tuft volume was calculated using Equation 1:

GV = (*β*/*κ*) xGA^3/2^ (1), where *β* = 1.38, the shape coefficient of spheres (the idealized shape of glomeruli), and *κ* = 1.1, the size distribution coefficient, and GA, glomerular area. The mesangial matrix expansion was defined as a periodic acid–Schiff–positive and nuclei-free area in the mesangium. Quantification of mesangial expansion was based on 20 glomeruli cut at the vascular pole per mouse in each group.

### Immunofluorescence

Kidney sections from these mice were prepared in an identical fashion. Immunostaining was performed using anti-WT-1 from Santa Cruz (sc-192) or anti-collagen IV antibody (Chemicon, ab756p) at 1:50 dilution. After staining, slides were mounted in Aqua Poly/Mount (Polysciences Inc.) and photographed under an AxioVision IIe microscope with a digital camera. Counting of podocytes and quantification of glomerular area and volume were performed using ImageJ and by the method standardized by Animal Models of Diabetic Complications Consortium^57^. ImageJ 1.26t software was used to measure the level of immunostaining in the glomeruli. First, the images were converted to 8-bit grayscale. Next, the glomerular region was selected for the measurement of area and integrated density. Next, the background intensity was measured by selecting three distinct areas in the background with no staining. The corrected optical density (COD) was determined using Equation 2: COD = ID – (*A* × MGV), where ID is the integrated density of the selected glomerular region, A is the area of the selected glomerular region, and MGV is the mean gray value of the background readings.

### TEM and morphometric studies

Tissues were fixed in 2.5% glutaraldehyde with 0.1 m sodium cacodylate (pH 7.4) for 72 h at 4 °C. Samples were further incubated with 2% osmium tetroxide and 0.1 m sodium cacodylate (pH 7.4) for 1 hr at room temperature. Ultrathin sections were stained with lead citrate and uranyl acetate and viewed on a Hitachi H7650 microscope. In brief, negatives were digitized, and images with a final magnitude of up to ×10,000 were obtained. ImageJ 1.26t software (National Institutes of Health, rsb.info.nih.gov) was used to measure the length of the peripheral GBM, and the number of slit pores overlying this GBM length was counted. The arithmetic mean of the foot process width (*W*_FP_) was calculated using Equation 3: *W*_FP_ = (*π*/4) × (*Σ*_GBM length_/*Σ*_slits_)*,* where Σ_slits_ indicates the total number of slits counted, *Σ*_GBM length_ indicates the total GBM length measured in one glomerulus, and *π*/4 is the correction factor for the random orientation by which the foot processes were sectioned^58^.

### mRNA sequencing

Mice glomeruli were isolated as previously reported^59^. Total RNA was extracted from glomeruli using Trizol method. Purified RNA underwent DNA digestion using RNase-free DNase set (79254, Qiagen). mRNA sequencing was performed at Beijing Genomics Institute.

### Cell culture

Immortalized human podocytes were obtained from Moin Saleem^60^. Cells were serum starved in 1% serum-containing medium for 12 h followed by treatment with the medium containing either normal glucose (5 mM, with 25 mM mannitol as high osmolarity control) or high glucose (30 mM) for the indicated time intervals.

### Transfection

Immortalized podocytes were transfected with 5 μg of empty vector or mouse PPP2cb using Viafect reagent (Promega, E4981). Forty-eight hours after transfection, cells were treated with or without 10 ng/ml of TNF-α for additional 24 h.

### DARTS assay

HEK293T cells were lysed in M-PER mammalian protein extraction buffer (78501 Thermo Fisher) with proteinase inhibitor (11836153001, Roche) and phosphatase inhibitors (50 mM NaF, 10 mM β-glycerophosphate, 5 mM sodium pyrophosphate, 2 mM Na_3_VO_4_). In all, 10× TNC buffer (500 mM Tris-HCl pH 8.0, 500 mM NaCl, 100 mM CaCl_2_) was added into cell lysate and protein concentration was measured using the Bradford assay (Bio-rad, #500-0006). The cell lysate was incubated with ATG (10 μM) at room temperature for 1 h. After incubation, cell lysates from ATG-treated or were subject to proteolysis at room temperature for 20 min with various dilutions of pronase (Roche, #10165921001) as indicated for western blotting. Lysate treated with 1:1000 dilution of pronase were used for mass spectrometry analysis. Proteins with enrichment ≥ 3 in ATG-treated sample compared to vehicle sample were considered as candidate ATG-binding proteins.

### Immunoprecipitation

PP2A alpha subunit tagged with FLAG was expressed in HEK293T cells, and PP2A alpha subunit was immunoprecipitated with anti-FLAG magnetic beads (Sigma-Aldrich, #M8823). Accompanied proteins were separated on dodecyl sulfate polyacrylamide gel electrophoresis (SDS-PAGE) gel and subjected to mass spectrometry analysis. Proteins with enrichment  ≥ 3-fold in PP2A alpha pull-down sample compared with control vector sample were considered as candidate PP2A-interacting proteins.

### Mass spectrometry analysis

MS analyses were performed after DARTS or immunoprecipitation assay. As these screening experiments were performed without replicates per experimental condition (*n* = 1 per sample), no statistical analysis was applied. For in-solution digestion of proteins, following steps were taken: the total protein concentration was determined using Branford Assay. Eight micrograms of protein solutions (DMSO and ATG) from each sample was in-solution digested by trypsin. In brief, the protein was reduced by 2 mM Dithiothreitol (DTT) at 55 °C for 30 min followed by 10 mM iodoacetamide (IAM) alkylation at room temperature for 10 min. Trypsin (Promega, Cat # V511A) was added in a ratio of 1:50 (trypsin:protein) and incubated at 37 °C overnight. The peptides were C18 desalted for LC-MS/MS analysis. For in-gel digestion, the protein samples were first separated on SDS-PAGE. After coomassie blue staining, the gel was cut into ~ 1 mm cubes and rinsed with 30% acetonitrile (ACN) in 50 mM NH4HCO3 until the coomassie blue stain completely removed. In all, 5 mM DTT was added to the gel for protein reduction at 55 °C for 30 min followed by the alkylation with 25 mM IAM. Trypsin digestion was performed at 37 °C for overnight. The resulting peptides were extracted and desalted with a C18 spin column (Thermo Fisher Scientific) for LC-MS/MS analysis. The peptides from either in-solution digestion or in-gel digestion were analyzed by LC-MS/MS on a U3000 Ultimate nano LC system coupled with Q Exactive MS instrument (Thermo Scientific). In brief, the peptides was resuspended in Solvent A (2% ACN in 0.1% FA) and separated on a C18 reversed phase nano column (Acclaim Pep RSLC 75 μm × 50 cm, 2 μm, 100 Å, Thermo Scientific) with a 185-min of binary gradient of Solvent A and Solvent B (85% ACN in 0.1% FA). The gradient is set as the following: 0 –10 min from 1% B to 5% B, 165 min to 30% B, 180 min to 50% B and 185 min to 95% B. The eluent peptides were directly introduced to mass spectrometry via Nanospray FlexTM ion source. The MS spectra was acquired in a positive mode with the spray voltage is 2.15 kV and the ion transfer tube temperature of 275 °C. The mass range is *m*/*z* 350–1700 with the resolution of 70,000 FWHM and AGC of 1E6 for MS scan. Fifteen most intensive ions with charge state between 2 and 5 were selected for MS/MS analysis with the dynamic exclusion of 60 s. Higher-energy Collisional Dissociation (HCD) was used for peptide fragmentation with the Collison Energy of 27%. The resolution for MS/MS analysis was 17,500 FWHM with AGC of 5.0E4. Isolation window was set to 2 *m*/*z* and maximum injection time for both MS1 and MS2 were 100 ms. The MS/MS spectra were searched against a SwissProt human database (20,223 sequences) using a local MASCOT (V.2.3) search engine on the Proteome Discoverer (V1.4, PD) platform with the MS tolerance of 10 ppm and fragment tolerance of 0.1 Da. Carbomidomethylation of cysteine was selected as fixed modification, whereas oxidation of methionine, phosphorylation of serine and threonine, and acetylation of N terminus of protein were selected as variable modification. Maximum missed cleavage of trypsin is 2. The false discovery rate for both proteins and peptides was less than 1%. For the protein relative quantitation, the spectra counting method was used. To avoid exaggerating the ratio from the small spectra counts, we arbitrarily added 2 in each spectra counts prior to ratio calculation in order to prevent division of 0, and to better reflect the fold change in protein levels. In particular, proteins with low spectral counts (e.g., 0, 1) would present as arbitrarily large fold changes owing to the small denominator. The exact number is empirical and technology dependent, and adding a small value (e.g., 2) is ideal and balanced, as it prevents inflation of fold changes without diluting the observed changes. Only the ratio changes over 2 folds were considered as changed. For phosphopeptides, the PhosphoRS node in PD was used to evaluate the probability of the phosphorylation site assignment. We used peptides with 75% or greater probability score and affected by ATG treatment for downstream validation and analysis.

### PP2A activity measurement

HEK293T cells and podocytes were treated with various concentration of ATG for 2 h. PP2A activity was measured according to the manufacturer’s protocol (R&D systems, DYC3309-2).

### DBN1 expression vectors

Human DBN1 (isoform E) was amplified using cDNA synthesized from human podocyte mRNA. FLAG peptide sequence was incorporated into the sense primer containing a terminal BamH1 site. The anti-sense primer contained a terminal EcoRV site. Primers used for amplification were: Forward, 5′-GGATCCATGGACTACAAAGACGATGACGACAAGGCCGGCGTCAGCTTCAGCG-3′; Reverse, 5′-GATATCCTAATCACCACCCTCGA-3′. PCR-amplified products were cloned into pGEM-T-vector (Promega A3600). cDNAs of DBN1 were released from T-vector with BamH1 and EcoRV restriction enzymes and inserted into BamH1 and Sma1-digested pNL4/3 lentiviral vector. DBN1 T335A and S337A mutations were generated by site-directed mutagenesis (Agilent 200523) using the following primers: Forward, 5′-CGGGCTCCGCGCGGGGATGGGAG-3′, Reverse, 5′-CTCCCATCCCCGCGCGGAGCCCG-3′ for T335A; Forward, 5'-GGAGTCAGACGGGGCCCGCGTGGGGATG-3', Reverse, 5'-CATCCCCACGCGGGCCCCGTCTGACTCC-3'. WT and mutant DBN1 constructs were confirmed by direct DNA sequencing.

### Western blot

Cells were lysed in M-PER mammalian protein extraction reagent (Thermo Fisher, NY) containing protease inhibitor and tyrosine and serine-threonine phosphorylation inhibitors. Protein was separated on SDA-PAGE and transferred to nitrocellulose membrane. For phospho-protein analysis, extracted proteins were separated on Phos-tag gel (Wako) according to the manufacturer’s protocol. Immunoblotted proteins were detected using specific antibodies: phospho-p65 (Abcam, ab28856), total p65 (Cell Signaling, 4764), PP2A (Abcam, ab168350), GAPDH (Cell Signaling, 2118), Drebrin (Cell Signaling, 5202), tubulin beta-4 (Novus Biologicals, NBP1-57005), total tubulin beta (Cell Signaling, 86298), and beta-actin (Sigma, A5136). All primary antibodies were used at 1:1000 dilution.

### Modeling of interactions between ATG and PP2A

The crystal structure for PP2A was retrieved from Research Collaboratory for Structural Bioinformatics (RCSB) Protein Data Bank (PDB ID: 2IAE), and the coordinates for ATG were sketched using ChemBioDraw software (PerkinElmer, Waltham) and PubChem (https://pubchem.ncbi.nlm.nih.gov/). Structures of PP2A and ATG were prepared for docking using the Molecular Operating Environment (MOE) 2013.08 (Chemical Computing Group Inc, Montreal, Canada). All protein chains present in the PDB structure was retained for docking except removing water molecules. Hydrogens were added to both the protein crystal structure and the ligand using MOE. Docking of ligands was first performed using MOE for initial identification of conformations^61^. After initial docking with MOE, docking poses were refined further by using a flexible receptor protocol.

### SPR

We used SPR to determine the real-time interaction between ATG and PP2A. All SPR experiments were performed by Affina Biotechnologies, Inc. (Valhalla, NY). PP2A protein from Abcam (Ab128577) at 50 μg/ml was dialyzed against 10 mM MES, pH 6.5 with 1 mM TCEP, and immobilized on SC80m chip (Xantec Bioanalytics, Germany) (10 min activation with 0.5 M EDC/0.25 M NHS in 0.1 M MES, pH5.5) at 1 μl/min to get immobilization signal of 10,000 RU. The surface was deactivated with 0.1 M ethanolamine, pH 8.3. Total theoretical Rmax for 1:1 ATG binding to PP2A is 66 RU. Samples were prepared as twofold dilution series with the starting concentration of 100 μM in the running buffer for (−) ATG, forskolin (Sigma-Aldrich) (3.13, 6.25, 12.5, 25, 50, 100 μM), and 2 μM in the running buffer for FTY720 (Sigma-Aldrich) (0.03, 0.06, 0.125, 0.25, 0.5, 1, and 2 μM). Running and sample buffer contained 25 mM HEPES, 0.005% Tween-20, 1 mM TCEP, 1% DMSO, and pH 7.4 at 25 °C. Flow and injections scheme consisted of 60 µl/min for 1 min followed by 2 min of dissociation. Double reference method was used for analysis. Reference channel was activated with EDC/NHS and deactivated with 1 M ethanolamine, pH 8.3. Measurements for selected concentrations for each compound were repeated twice and the duplicates are shown in the Results. Sensorgram response data were analyzed by using the BIA evaluation kinetics software, and the equilibrium binding and disassociation constants were calculated.

### Podocyte migration

In total, 1 × 10^4^ differentiated human podocytes were seeded onto Transwell culture inserts (pore size 5 μm, Costar Corporation) and allowed to migrate for 24 h in the medium with or without ATG (10 μM) in either normal glucose (5 μM glucose + 25 μM mannitol) or high glucose (30 μM glucose) conditions. Non-migratory podocytes were removed from the upper surface of the membrane. Migrated podocytes were fixed with 4% paraformaldehyde and stained with 0.1% crystal violet solution. The number of migrated cells in the center of a visual field was counted using phase-contrast microscope with a ×20 objective.

### Podocyte adhesion

Differentiated human podocytes were treated with either 5% DMSO or 10 μM ATG in normal or high glucose conditions for 24 h. Podocytes were then trypsinized and 1 × 10^4^ podocytes were plated on fibronectin (10 μg/ml)-coated 24-well plates in the same condition of medium for 1 h. To quantify adhesive podocytes, free-floating podocytes were washed off by gentle aspiration. To quantify total podocytes, unwashed plates were centrifuged at 200 g for 5 min at room temperature. Podocytes were fixed with 4% paraformaldehyde, stained by 0.1% crystal violet. Absorbance was measured at 570 nm. Adhesion ratio was calculated by dividing OD of total podocytes by OD of adhesive podocytes of the same condition.

### High-content image analysis

We used previously established high-content image acquisition, processing and analysis techniques to quantitatively determine morphological parameters of immortalized human podocytes in a high-throughput manner^62^. In brief, cells were plated on black glass-bottom 96-well imaging plates (Nunc) at a concentration of 1500 cells per cm^2^ and cultured at 37 °C for 10 days. They were then treated with normal or high-glucose conditions with or without ATG for 24 h, fixed with 4% paraformaldehyde and immunostained for paxillin (using mouse anti-paxillin and AlexaFluor-488 anti-mouse antibodies from Thermo) and F-actin (using AlexaFluor-568 phalloidin from Thermo). Cells were imaged on a Leica DMi8 Infinity TIRF microscope using an oil-immersion × 63 TIRF objective whereby each well is tile scanned at an effective resolution of 0.13 μm per pixel. Nuclear (DAPI) images were captured as regular widefield images using Lumencor Spectra III LED illumination at 390 nm excitation. Paxillin images were captured using 488 nm excitation with (full) internal reflection with 90 nm z-depth while F-actin stress fiber images were captured using 561 nm laser with partial internal reflection with 250 nm z-depth. Images were then processed using custom scripts as previously described^18^ to extract cellular/nuclear area, perimeter and aspect ratio, stress fiber length, and focal adhesion area. Values from different groups were compared using two-way analysis of variance (ANOVA) with post hoc Tukey test.

### DBN1 and F-actin binding

WT and T335A DBN1 were expressed in differentiated human podocytes. DBN1 was pulled down with anti-FLAG antibody. DBN1 and actin were immunoblotted.

### Quantitative PCR

Total RNA was extracted from kidney cortices by using TRIzol (Thermo Fisher Scientific, Waltham, MA). First-strand cDNA was prepared from total RNA (2.0 μg) using the Superscript III first-strand synthesis kit (Thermo Fisher Scientific, Waltham, MA) and cDNA (1 μl) was amplified in triplicate using SYBR GreenER qPCR Supermix (Thermo Fisher Scientific, Waltham, MA) on an ABI PRISM 7900HT (Applied Biosystems, Foster City, CA). The primers used are listed in Supplementary Table 12. Data were normalized to *Gapdh* and presented as fold increase compared with RNA isolated from control animals using the 2^-∆∆CT^ method.

### Statistical analysis

Data are expressed as mean ± SD. The unpaired *t* test was used for comparisons between two groups and ANOVA followed by Tukey’s post hoc analysis was used for comparisons between multiple groups using the GraphPad Prism software. *P* < 0.05 was considered statistically significant.

### Reporting summary

Further information on experimental design is available in the Nature Research Reporting Summary linked to this paper.

## Supplementary information

Supplementary Information

Reporting Summary

Source Data

## Data Availability

RNA-seq data that support the findings of this study have been deposited in the Gene Expression Omnibus (GEO) repository under the accession # GSE134327. The mass spectrometry proteomic data set is deposited to the ProteomeXchange Consortium via the Proteomics IDEntifications (PRIDE) partner repository with the data set identifier PXD014976 and 10.6019/PXD014976. All other data generated or analyzed during this study are included in this published article (and its supplementary information files). The source data underlying Figs. 1b, 1d, 2b, 2d, 3a, 3c–f, 4a–d, 5a–f, 6b, 6d, 7b, 7d, 8b, 8e, and Supplementary Figs. 1, 2, 4, and 7 are provided as a Source Data file. All data are also available from the corresponding authors upon reasonable request.
