# Supplementary material for: Arctigenin attenuates diabetic kidney disease through the activation of PP2A in podocytes
Source: Nat Commun. 2019 Oct 4;10:4523. doi: 10.1038/s41467-019-12433-w (PMC6778111; doi:10.1038/s41467-019-12433-w)
Supplement: Supplementary file 1 — Supplementary Information [file 41467_2019_12433_MOESM1_ESM.pdf]

Arctigenin attenuates diabetic kidney disease through the activation of PP2A in podocytes

Zhong Y et al.

Supplementary Table of Contents

Supplementary Figures 1-15

Supplementary Tables 1-12

Supplemental Figures

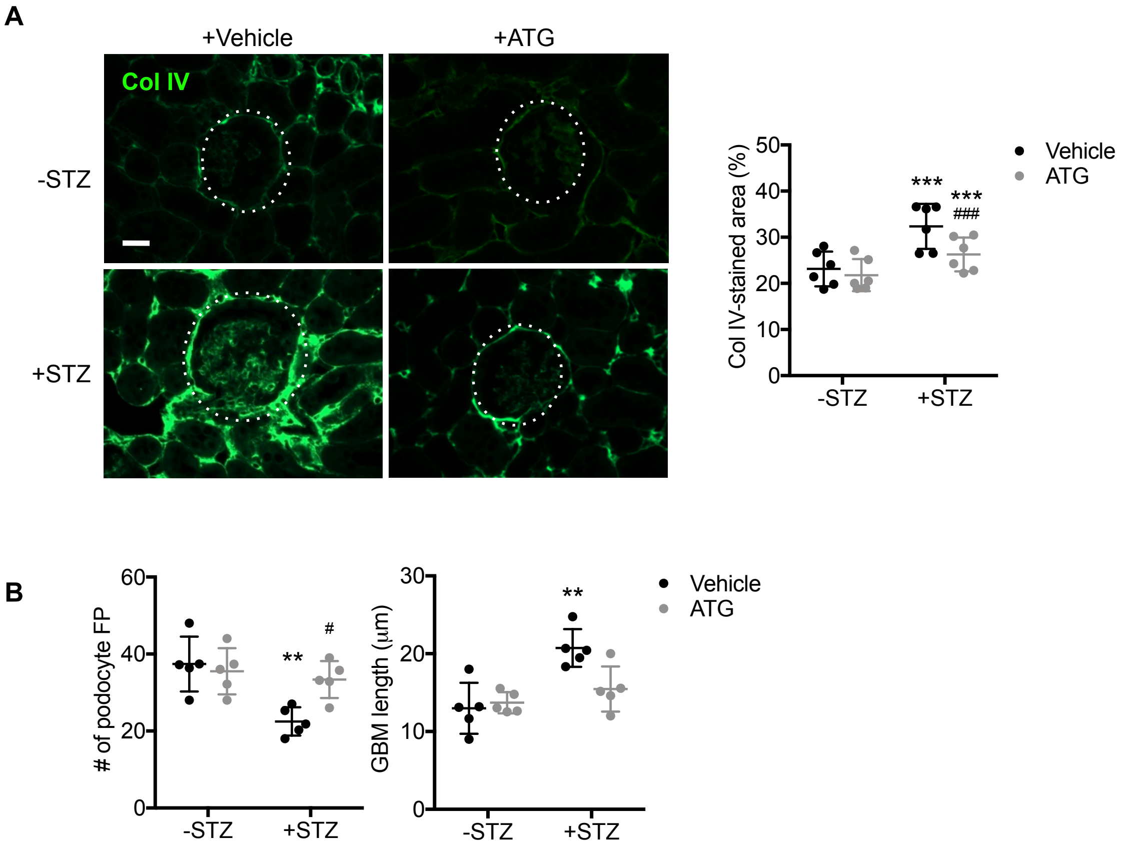

Supp. Figure 1: ATG treatment reduces mesangial expansion and ultra-structural changes in the diabetic eNOS$^{-/-}$ mice. (A) Representative images of collagen IV immunofluorescence in the glomeruli of diabetic and control eNOS$^{-/-}$ mice. Glomeruli are outlined with a white dotted circles; scale bar, 20μm. Quantification of glomerular collagen IV immunostaining is shown on the right. ****P<0.0001 when compared with nondiabetic controls; $^{\#\#\#\#}$P<0.0001 when compared with vehicle-treated diabetic eNOS$^{-/-}$ mice; n=6 per group. (B) Quantification of podocyte foot processes (FP) and glomerular basement membrane lengths used to calculate average podocyte FP effacement in Figure 2A is shown. **P<0.01 when compared with nondiabetic controls; $^{\#}$P<0.05 when compared with vehicle-treatment. n=6 in each group. The data are represented as mean ± SD. Source data are provided as a Source Data file.

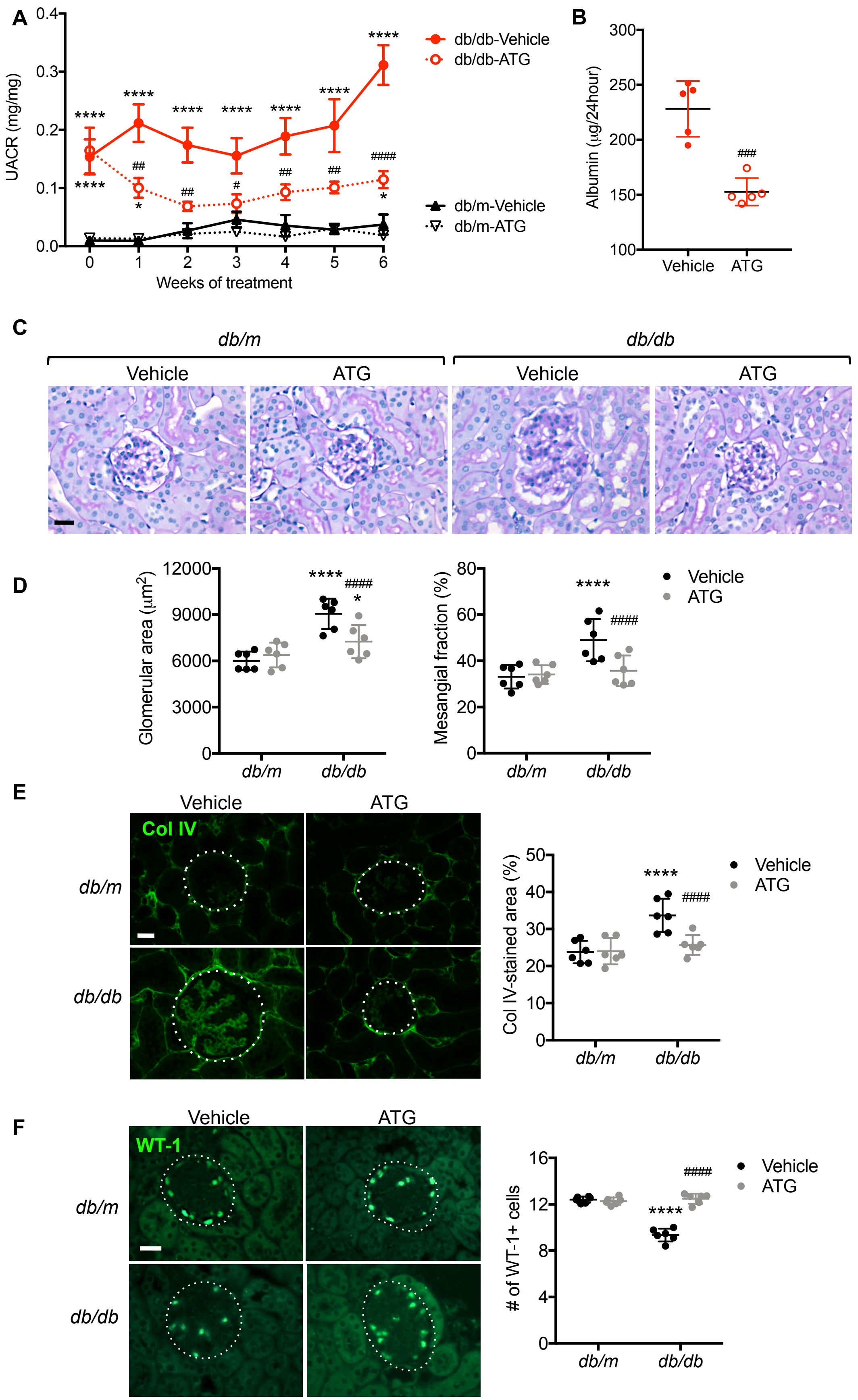

Supp. Figure 2: The effects of ATG treatment on *db/db* diabetic mice. (A) Analysis of urinary albumin-to-creatinine ratio (UACR). ****P<0.0001 when compared with nondiabetic controls; ####P<0.0001 vs. vehicle-treated *db/db* mice; n= 5-6 mice per group. (B) Albumin in 24-hour collected urines after 6 weeks of ATG treatment in *db/db* mice. ###P<0.001 vs. vehicle-treated mice. (C) Representative images of periodic acid-Schiff (PAS)-stained kidneys. Scale bar, 20µm. (D) Quantification of glomerular area and mesangial fraction in diabetic and control mice. *P<0.05 and ****P<0.0001 vs. nondiabetic controls; ####P<0.0001 vs. vehicle-treated *db/db* mice. (E) Representative images and quantification of collagen IV immunofluorescence in the glomeruli of diabetic and control eNOS$^{-/-}$ mice. Glomeruli are outlined with a white dotted circles; scale bar, 20µm (F) Representative images of WT1+ immunofluorescence and quantification in the glomeruli of diabetic and control mice. Scale bar, 20µm. ****P<0.0001 vs. nondiabetic controls; ####P<0.0001 vs. vehicle-treated diabetic mice. The data are represented as mean ± SD. Source data are provided as a Source Data file.

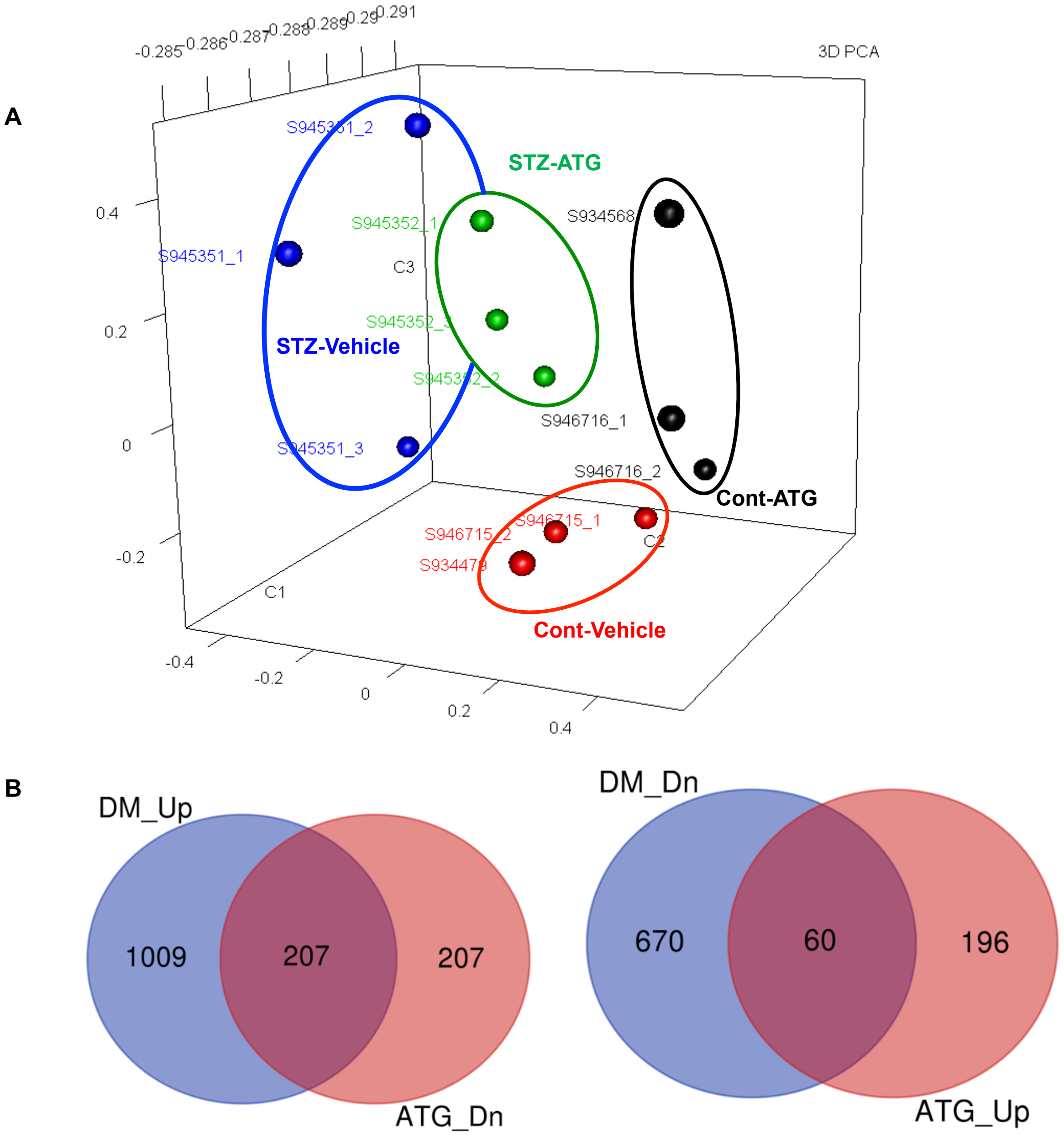

Supp. Figure 3: RNA-seq data from isolated mouse glomeruli of diabetic and control eNOS$^{-/-}$ mice. RNA-sequencing was performed with isolated glomeruli from diabetic (STZ) or control (Cont) mice treated with ATG or vehicle (n=3 in each group). (A) Principal component analysis. (B) Venn-diagrams showing the number of differentially expressed genes (DEGs) in the diabetic mice that were upregulated (DM_Up) and downregulated by ATG (ATG_Dn) and DEGs that were downregulated in the diabetic mice (DM_Dn) and upregulated by ATG (ATG_Up). (C) Heatmap of top 50 DEGs that were reversed by ATG treatment. (D) Real-time PCR validation of genes in the cell adhesion and actin regulation pathways in isolated glomeruli of diabetic and control mice. *P<0.05, **P<0.01, and ****P<0.0001 vs. non-diabetic controls; $^{\#\#}$P<0.01 and $^{\#\#\#\#}$P<0.0001 vs. vehicle-treated diabetic eNOS$^{-/-}$ mice; n=6 per group.

Supp. Figure 3 cont.

C

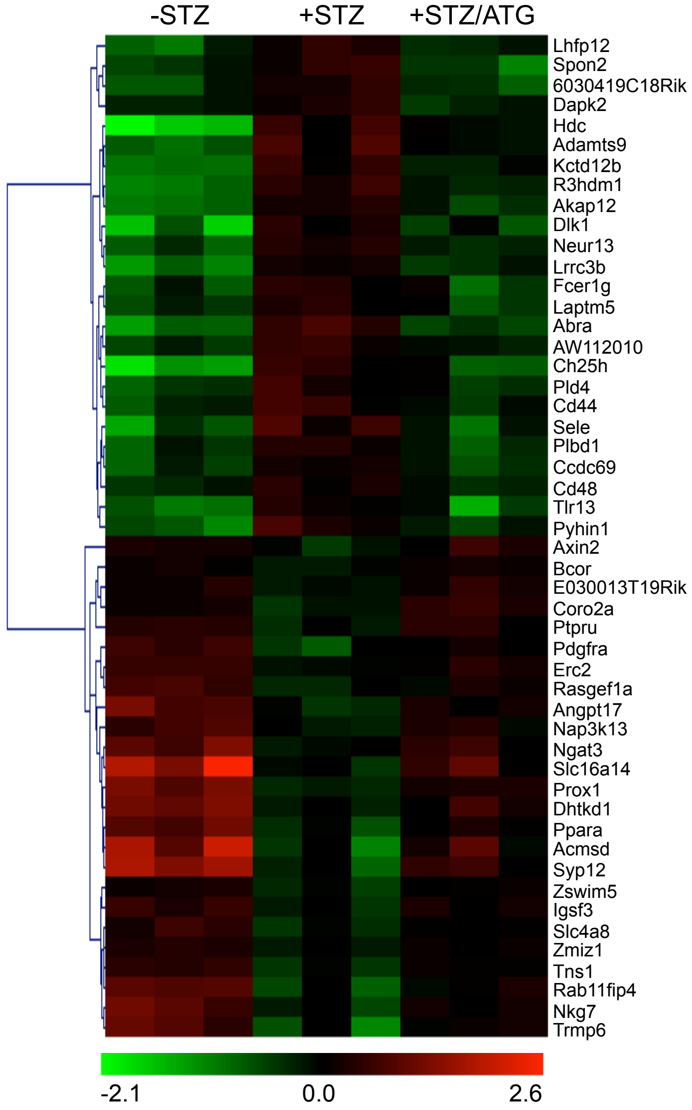

D

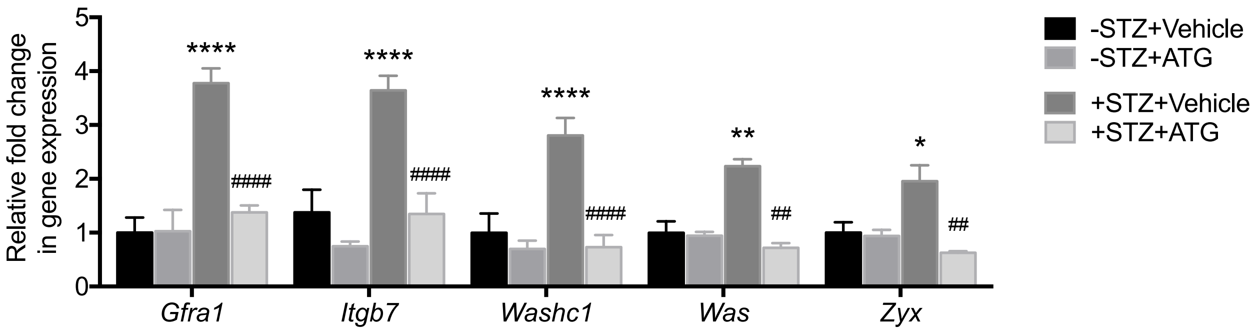

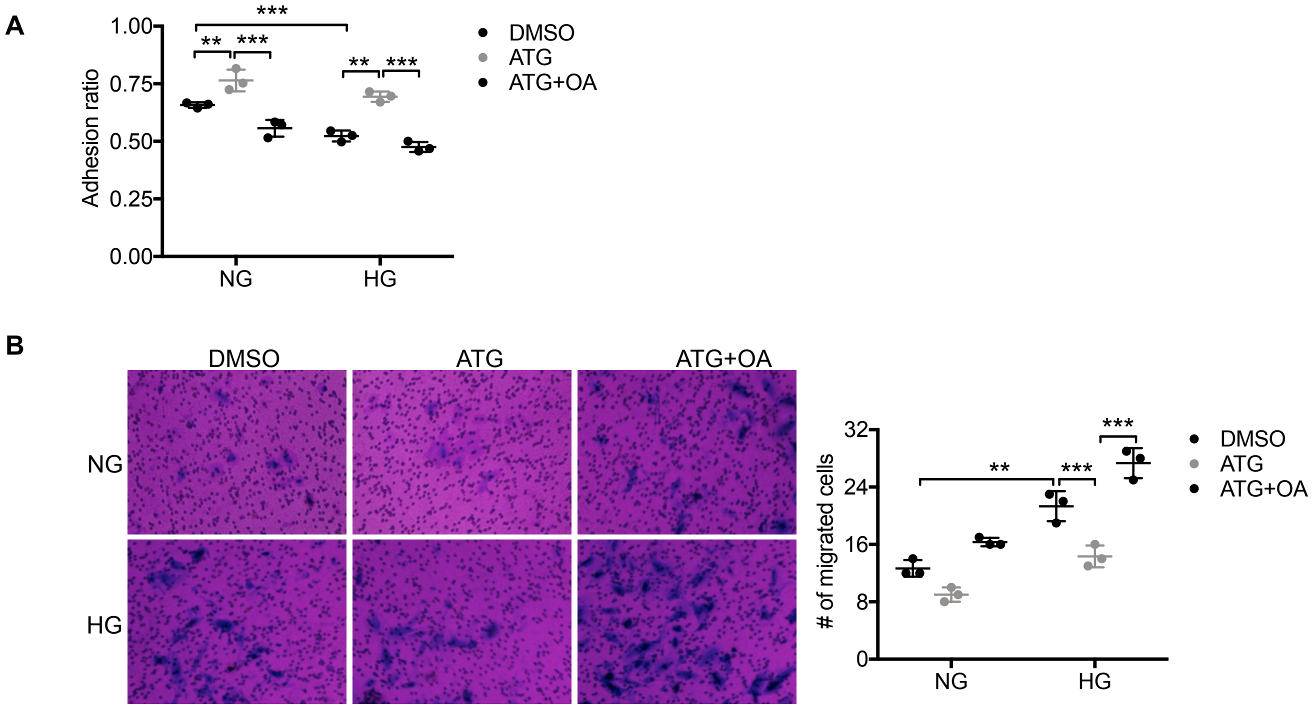

Supp. Figure 4: Okadaic acid abrogates the effects of ATG in podocyte adhesion and migration. (A) Quantification of adhesion of immortalized human podocytes cultured in normal (NG) or high glucose (HG) condition. DMSO vehicle, ATG (10μm), or ATG and okadaic acid (OA, 10nM) were added for 24 hours. Podocytes adhesion is expressed as ratio of adhered podocytes to total podocytes. Average ratio is shown for 3 experiments. (B) Representative images and quantification of migrated podocytes treated with DMSO vehicle or ATG in normal or high glucose medium. NG, normal glucose; HG, high glucose. **P<0.01 and ***P<0.001 when compared between indicated groups. Average number of cells is shown for 3 experiments. The data are represented as mean ± SD. Source data are provided as a Source Data file.

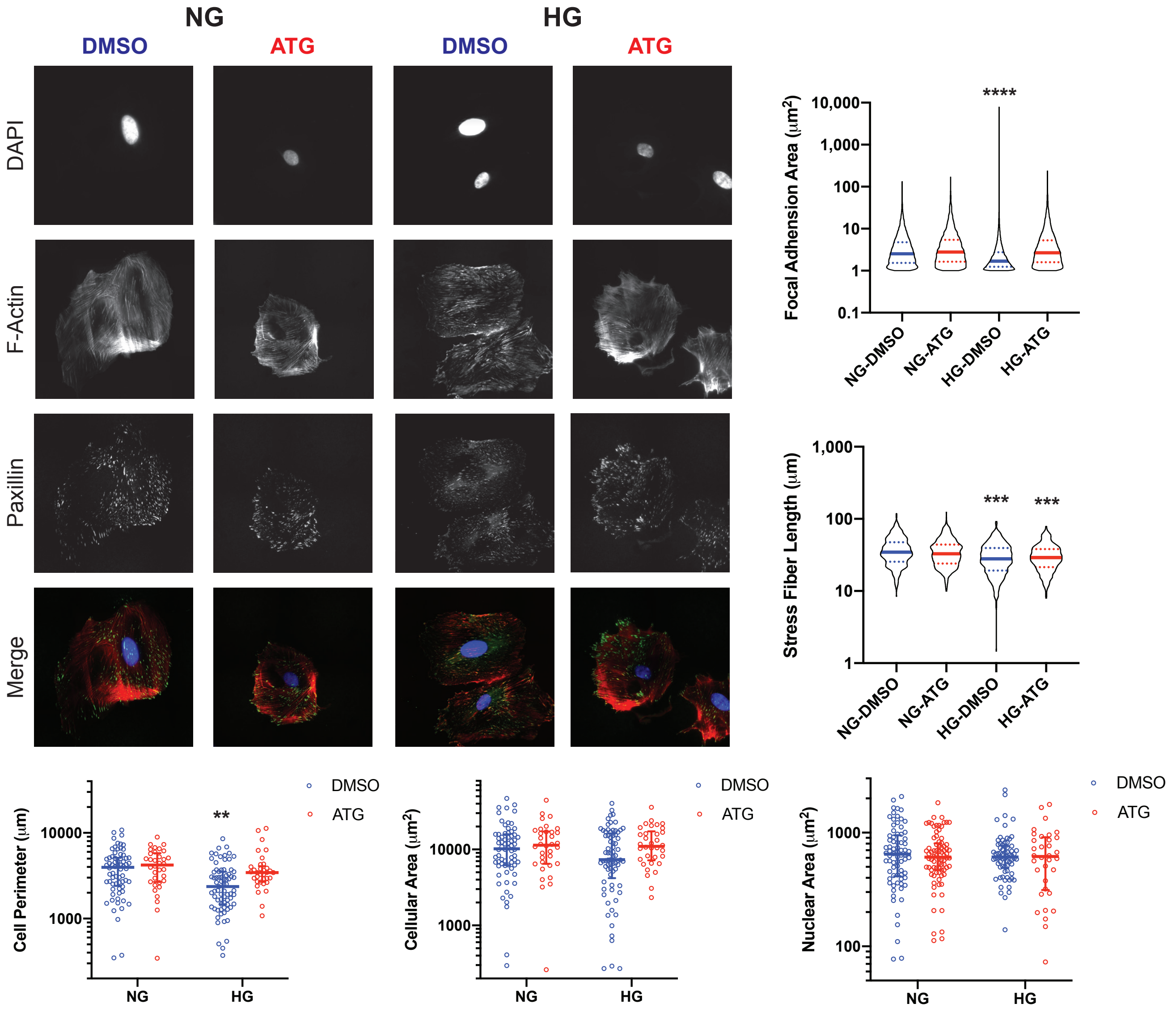

Supp. Figure 5: ATG enhances podocyte adhesion and morphology. Representative DAPI (blue), F-actin (red) and paxillin (green) images of immortalized human podocytes cultured under normal glucose (NG) or high glucose (HG) and with ATG (10μm) or with vehicle control (DMSO) for 24 hours. ATG reversed the significant reduction in focal adhesion area and cell perimeter under high-glucose conditions. While it increased both cellular area and stress fiber length in HG as well, the effect did not reach significance. Nuclear area was unaffected by high glucose or ATG treatment. $^{**}P<0.01$ and $^{****}P<0.0001$ when compared between indicated groups. The data are represented as mean ± SD.

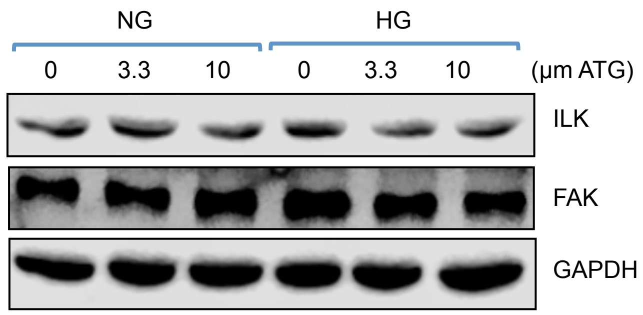

Supp. Figure 6: ILK and FAK protein levels are unaffected by ATG treatment. Western blot analysis of ILK and FAK levels in podocytes treated with increasing concentrations of ATG.

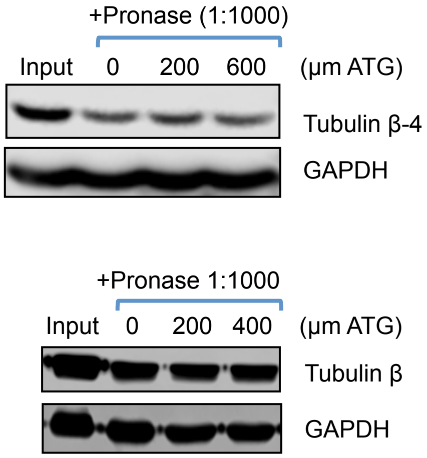

Supp. Figure 7: ATG does not bind to tubulin. (A) DARTS assay was performed to test the binding of ATG to tubulin in HEK293T cells. HEK293T cell lysates were pre-incubated with various concentrations of ATG as indicated at 25°C for 1 hour prior to digestion with pronase (0 or 1:1000 dilution) for 20 minutes. Lysates were then probed for antibody specific for tubulin β-4 (Novus Biologicals, #NBP1-57005; raised against a.a.86-135 of beta isoform-4) or total tubulin β (Cell signaling #86298; raised against a.a. residues near proline 32, recognizing all tubulin beta isoforms). GAPDH was used as a loading control.

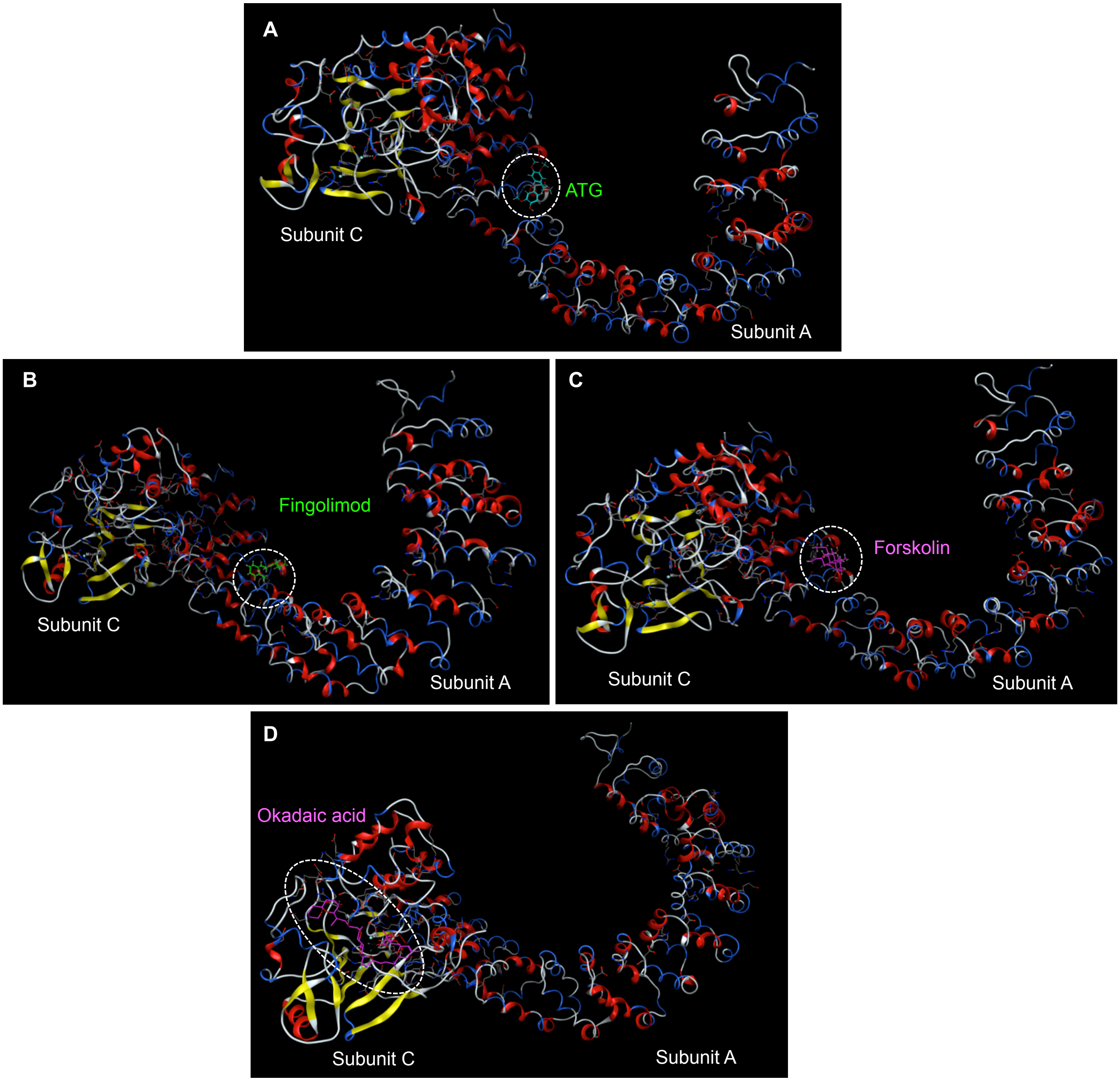

Supp. Figure 8: Comparison of predicted binding site of ATG (top panel), PP2A agonists fingolimod and forskolin (middle panels), and PP2A inhibitor okadaic acid (bottom panel) with PP2A. Positions of the binding sites are outlined in dotted circles.

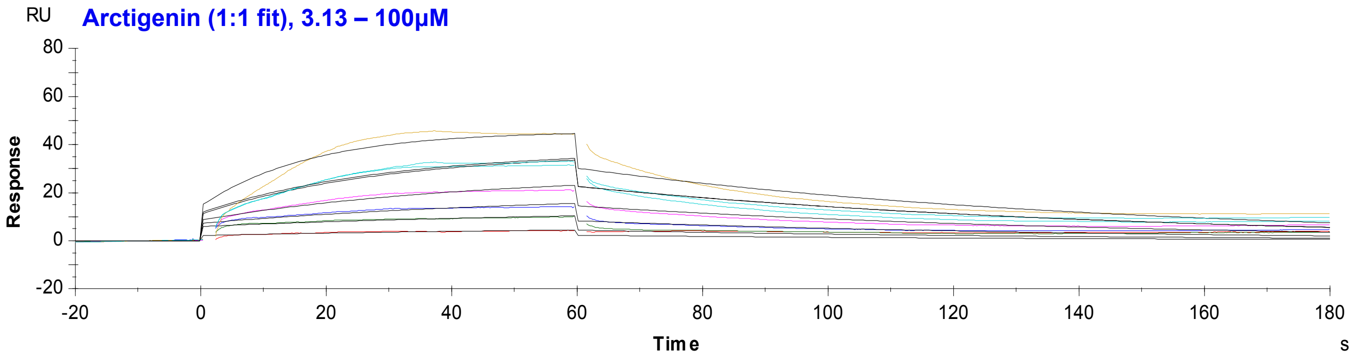

ka (1/Ms)	kd (1/s)	KD (M)	RI (RU)	Chi² (RU²)
456.7	0.01164	2.549E-5	39.16	2.98

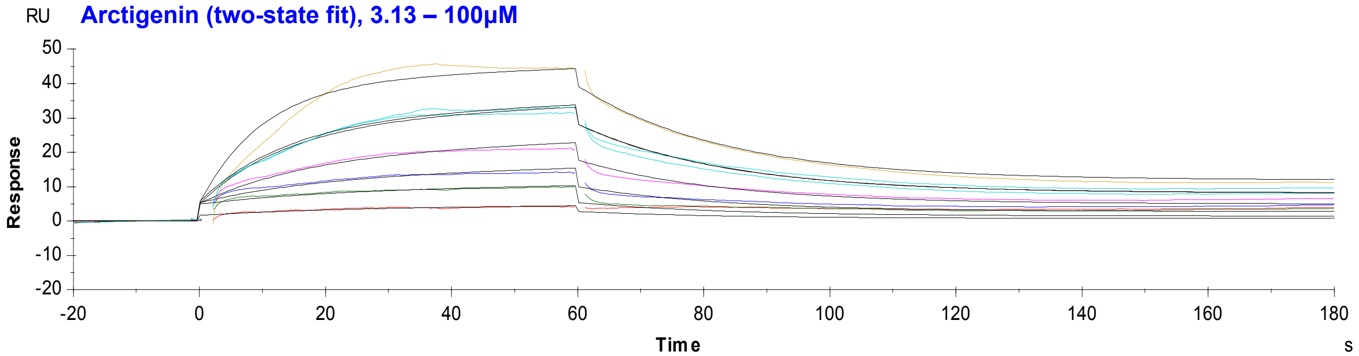

ka1 (1/Ms)	kd1 (1/s)	ka2 (1/s)	kd2 (1/s)	KD (M)	Rmax (RU)	Chi² (RU²)
594.8	0.03800	0.005015	2.610E-5	3.308E-7	60.22	1.79

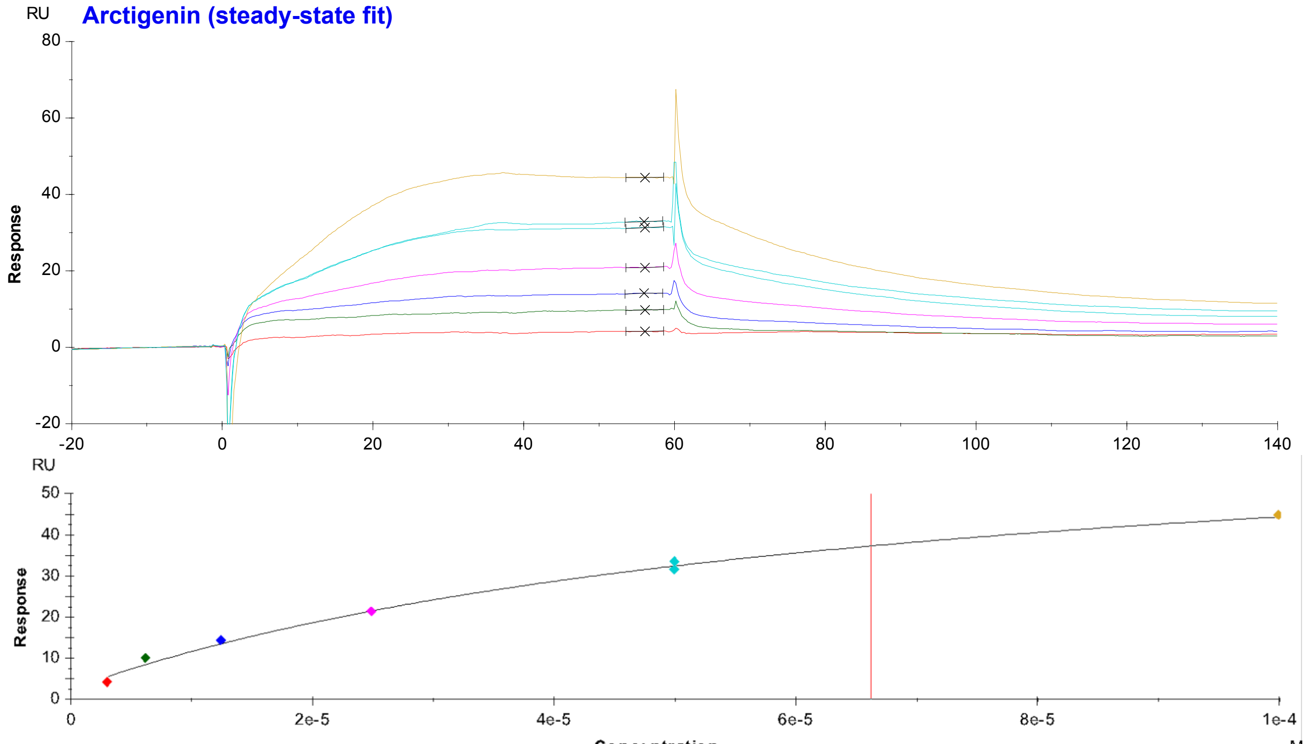

KD (M)	Rmax (RU)	Chi² (RU²)
6.620E-5	69.55	2.458

Supplementary Figure 9: **SPR assay confirms the binding of ATG to PP2A.** Analysis of binding of ATG to immobilized PP2A protein using 3 different models of fit. Parameters of each model is shown below.

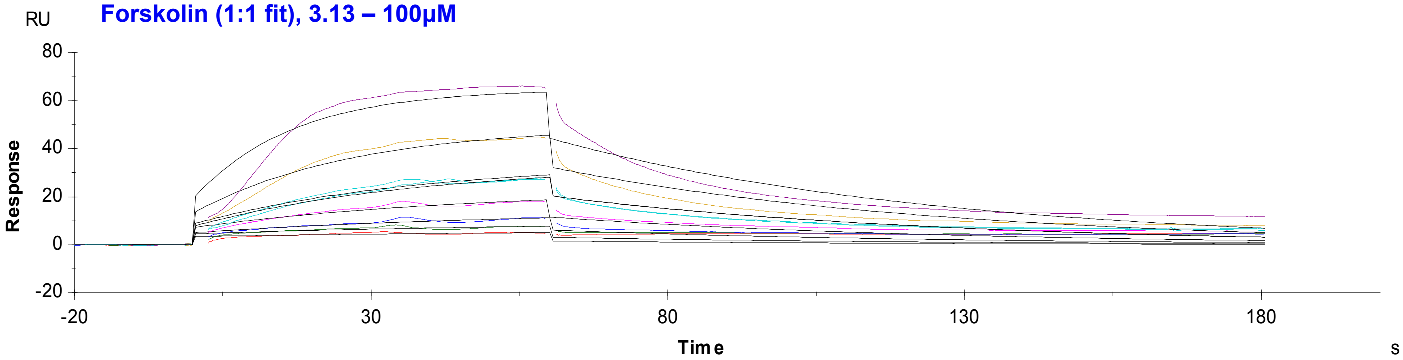

ka (1/Ms)	kd (1/s)	KD (M)	RI (RU)	Chi² (RU²)
220.4	0.01539	6.983E-5	61.61	5.71

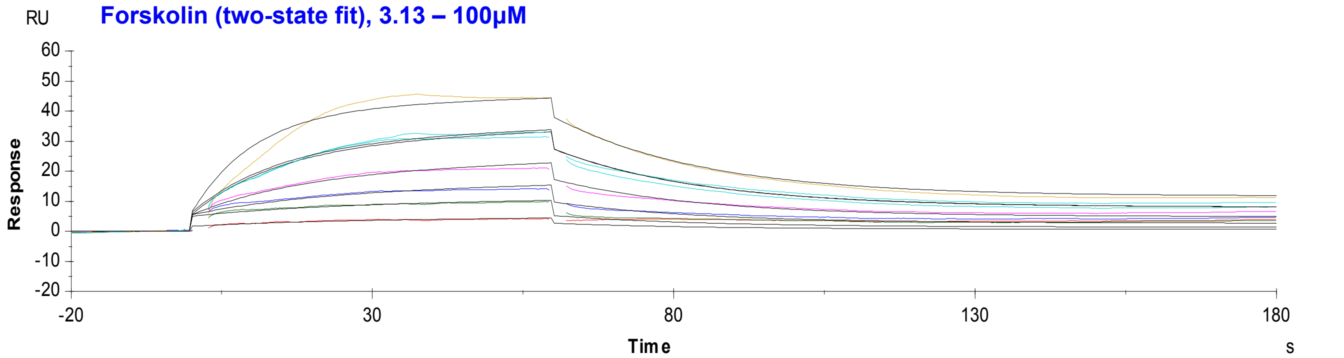

ka1 (1/Ms)	kd1 (1/s)	ka2 (1/s)	kd2 (1/s)	KD (M)	Rmax (RU)	Chi² (RU²)
584.8	0.03578	0.005008	3.951E-7	4.827E-9	57.75	1.71

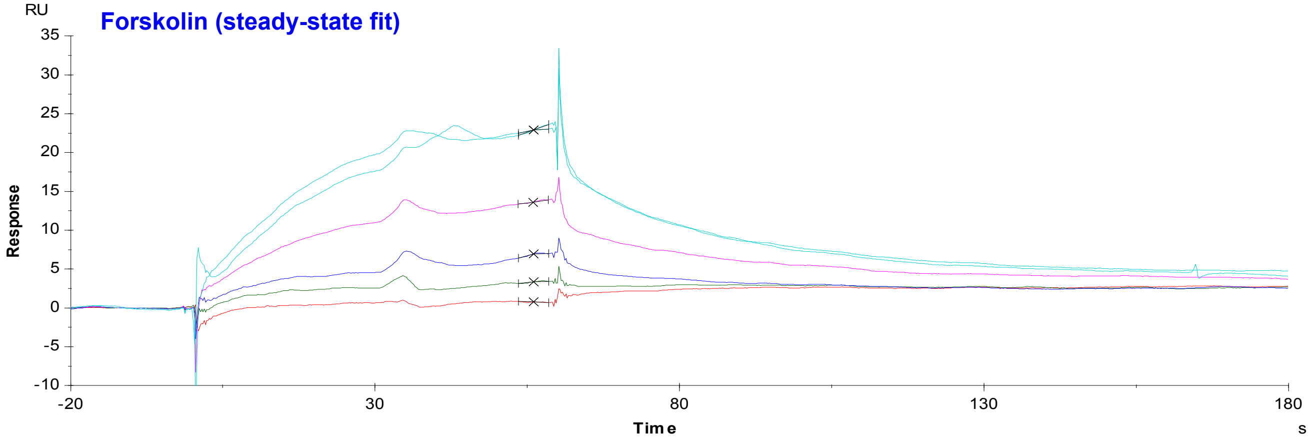

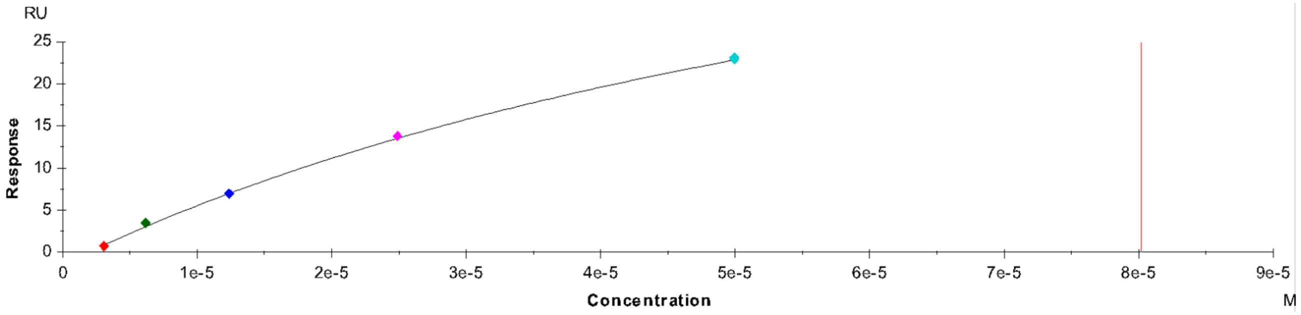

KD (M)	Rmax (RU)	Chi² (RU²)
8.018E-5	63.51	0.0445

Supplementary Figure 10: **Binding of forskolin to PP2A.** Analysis of binding of forskolin to immobilized PP2A protein using 3 different models of fit. Parameters of each model is shown below.

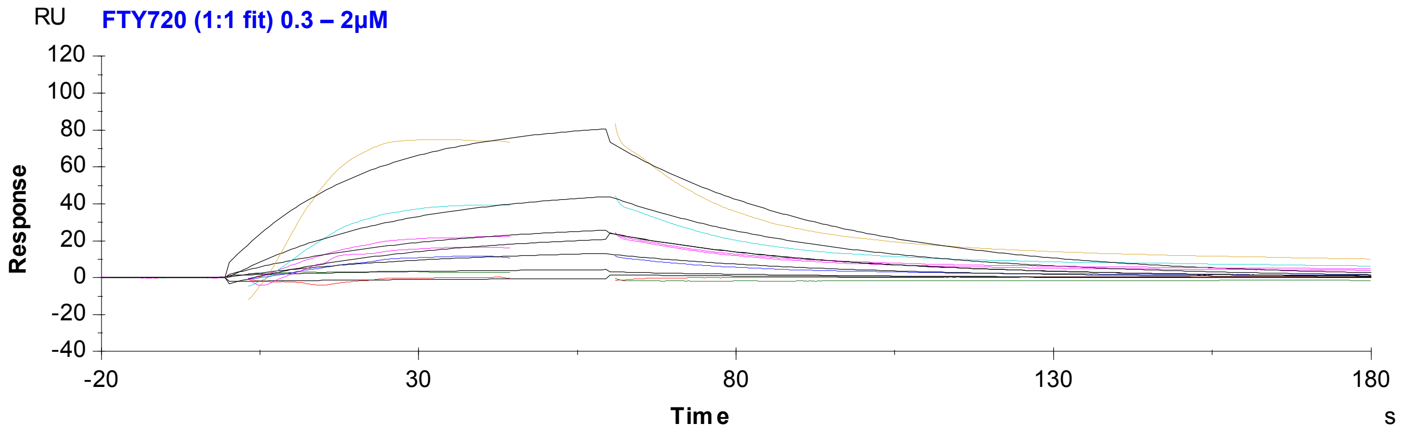

ka (1/Ms)	kd (1/s)	KD (M)	RI (RU)	Chi² (RU²)
9633	0.02760	2.866E-6	190.0	9.43

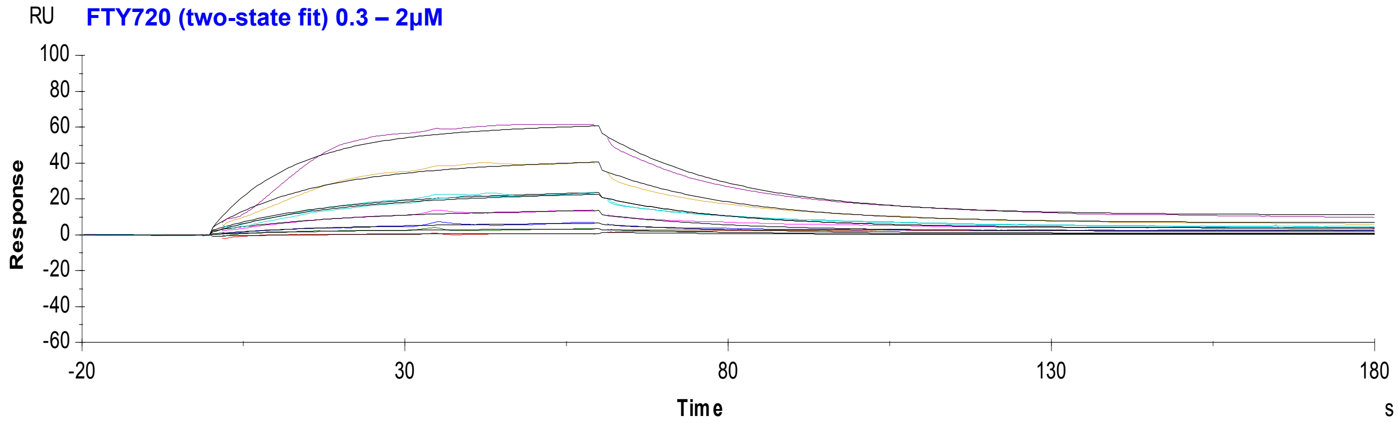

ka1 (1/Ms)	kd1 (1/s)	ka2 (1/s)	kd2 (1/s)	KD (M)	Rmax (RU)	Chi² (RU²)
182.2	0.04469	0.003158	7.838E-6	6.072E-7	122.2	1.89

Supplementary Figure 11: **Binding of fingolimod (FTY720) to PP2A.** Analysis of binding of FTY720 to immobilized PP2A protein using 3 different models of fit. Parameters of each model is shown below.

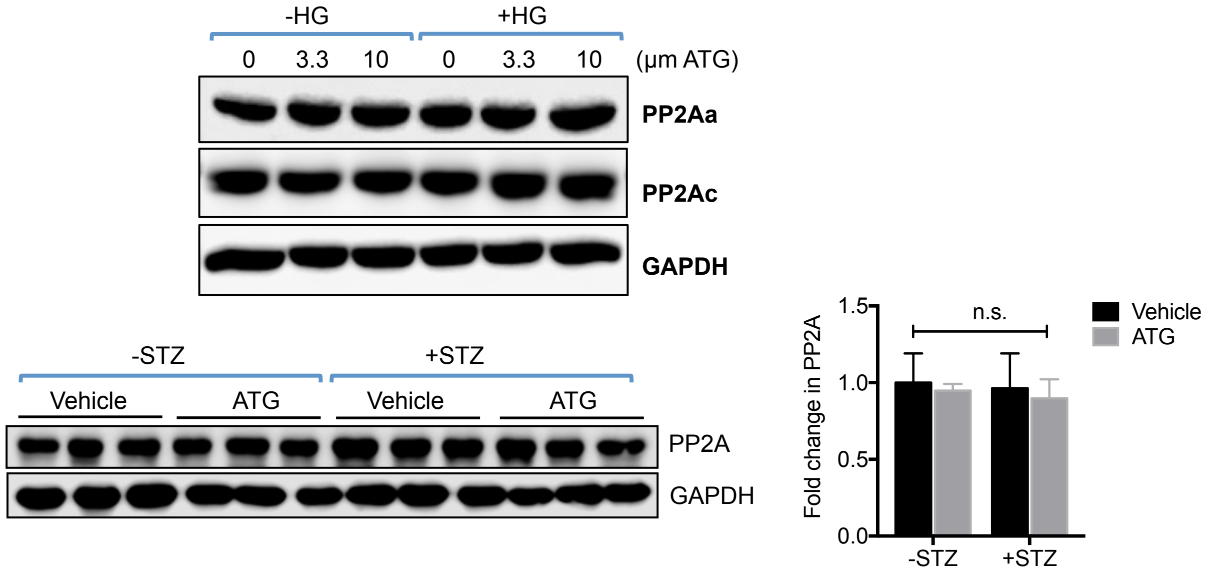

Supplementary Figure 12: ATG treatment does not alter the expression levels of PP2A. (A) Western blot showing similar levels of PP2A subunits A and C (PP2Aa and PP2Ac) in the cultured podocytes exposed to normal or high glucose conditions (± varying doses of ATG as indicated). (B) Level of PP2A is unchanged in the kidney glomeruli of control and diabetic mice with or without ATG treatment. Quantification of PP2A normalized to GAPDH is shown on the right. n=3 per group. The data are represented as mean ± SD. Source data are provided as a Source Data file.

DBN1 Expression in Lindenmeyer Normal Tissue Panel

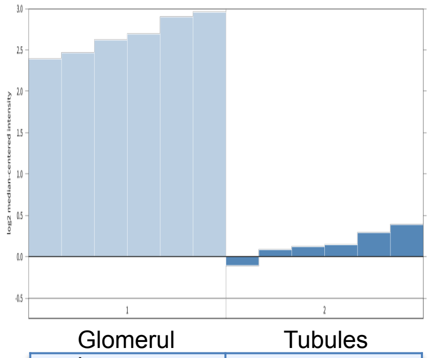

P-value:	0.003
t-Test:	-5.376
Fold Change:	-2.581

CKAP4 Expression in Lindenmeyer Normal Tissue Panel

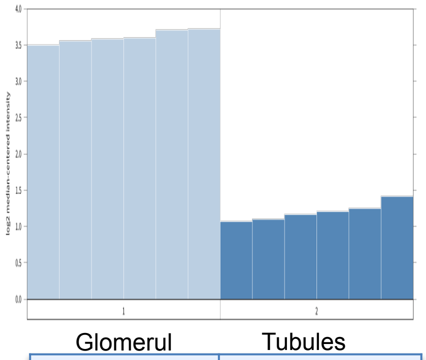

P-value:	1.24E-11
t-Test:	-38.782
Fold Change:	-5.319

DBN1 Expression in kidneys of healthy living donors (HLD) vs. DKD patients

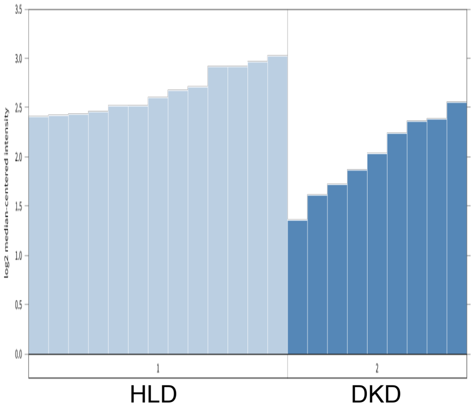

P-value:	5.36E-4
t-Test:	-4.333
Fold Change:	-1.564

CKAP4 Expression in kidneys of healthy living donors (HLD) vs. DKD patients

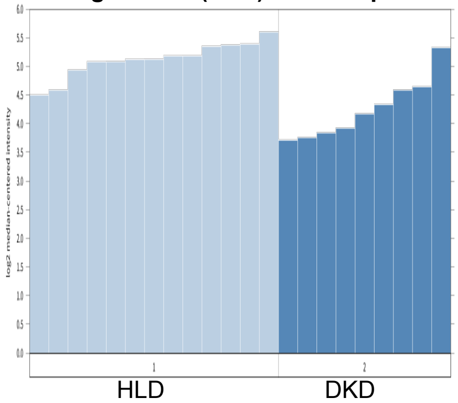

P-value:	4.52E-4
t-Test:	-4.401
Fold Change:	-1.815

Supp. Figure 13: Expression of DBN1 and CKAP4 mRNAs in human kidneys. Nephroseq dataset (nephroseq.org) showing the expression levels of DBN1 and CKAP4 in normal tissue (top panels) and in diabetic kidney disease (DKD) compared to healthy living donors (HLD) (bottom panels).

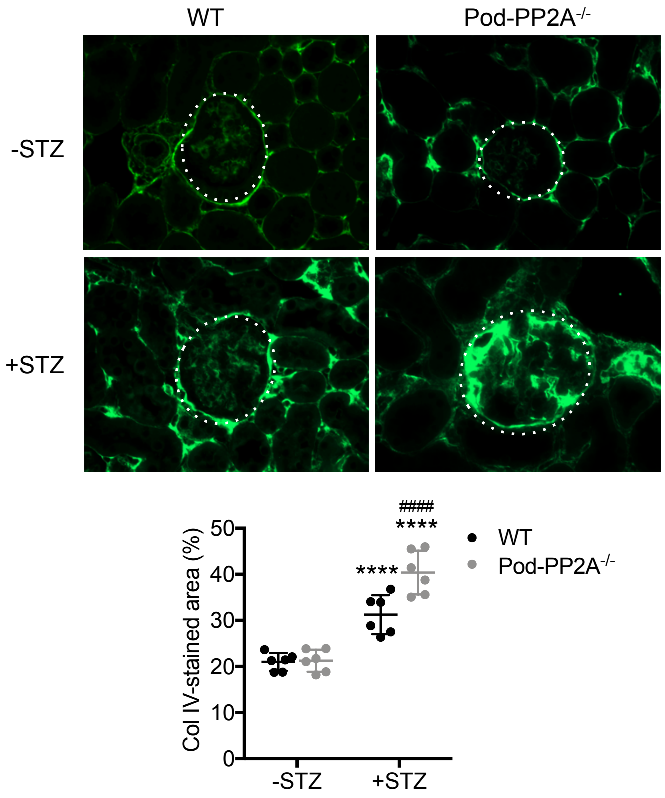

Supp. Figure 14: Collagen IV immunofluorescence of diabetic WT and Pod-PP2A$^{-/-}$ mice. Representative images of collagen IV immunofluorescence in the glomeruli of diabetic and control eNOS$^{-/-}$ mice. Glomeruli are outlined with a white dotted circles. Quantification of glomerular collagen IV immunostaining is shown below. ****P<0.0001 vs. respective nondiabetic mice; $^{\#\#\#\#}$P<0.0001 vs. WT diabetic mice; n=6. The data are represented as mean ± SD.

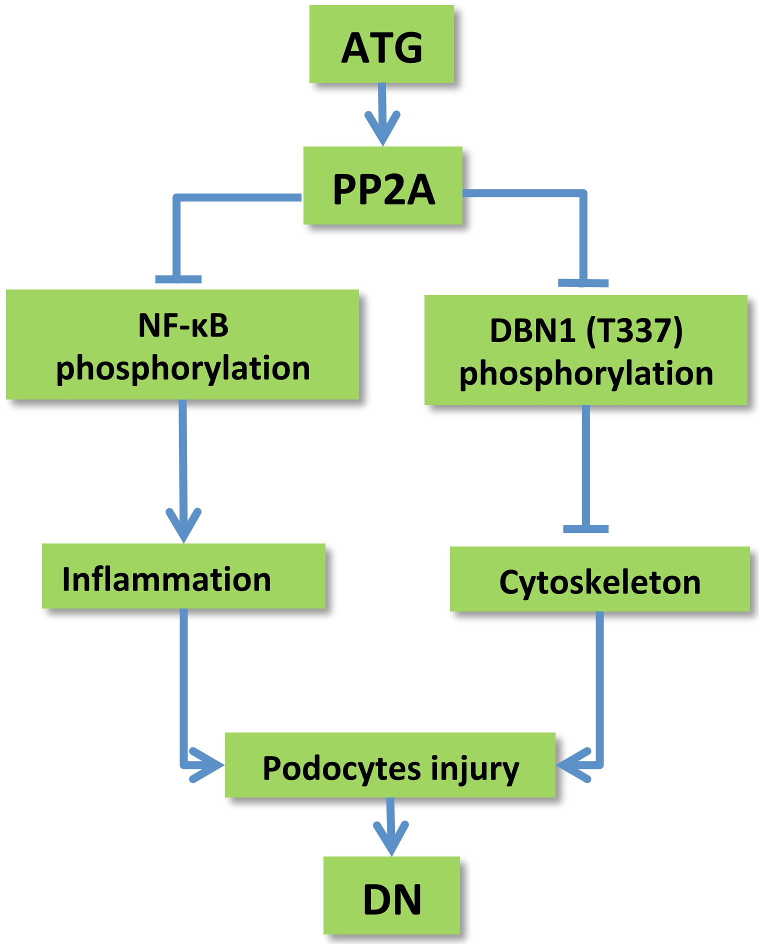

Supplementary Figure 15: Schematics of PP2A-mediated renoprotection of podocyte injury in DN through ATG.

Supplementary Tables

	Treatment groups	Baseline	Week 0	Week 2	Week 4	Week 6	Week 8
**-STZ**	Vehicle	94±8	92±9	94±1	95±9	92±6	103±7
	ATG	92±4	97±5	90±2	92±6	93±4	98±15
**+STZ**	Vehicle	94.7±9.1	448±61***	475±23***	360±89***	396±32***	406±13***
	ATG	113±11	418±54***	414±18***	397±27***	375±32***	429±45***

Supp. Table 1: Blood glucose levels in control and in diabetic eNOS$^{-/-}$ mice. Baseline values refers to levels prior to diabetes induction. Weeks indicate the number of weeks following ATG or vehicle treatment. ***P<0.001 when compared to respective non-diabetic mice. n=6 mice in each group.

	**-STZ**		**+STZ**	
**Treatment groups**	Vehicle	ATG	Vehicle	ATG
**BP (mmHg)**	119.6±4.7	123.4±4.5	138.4±2.3***	136.1±5.4**
**Total cholesterol (mg/dL)**	91.4±8.6	79.2±10.4	148.8±27.6**	145.5±24.1**
**Triglycerides (mg/dL)**	41.5±8.8	42.4±5.8	89.5±15.6**	91.8±8.1**

Supp. Table 2: Physiological parameters of control and in diabetic eNOS$^{-/-}$ mice at 18 weeks post diabetes induction. **P<0.01 and ***P<0.001 when compared to respective non-diabetic mice. n=5 mice in each group.

	**-STZ**		**+STZ**	
**Treatment groups**	Vehicle	ATG	Vehicle	ATG
**Kidney/BW (mg/g)**	8.0±0.5	8.2±0.6	12.7±0.7***	9.0±0.8$^{\#\#}$

Supp. Table 3: kidney to body weight (BW) ratio of control and in diabetic eNOS$^{-/-}$ mice at 18 weeks post diabetes induction. ***P<0.001 when compared to respective non-diabetic mice; and $^{\#\#}$P<0.01 when compared to vehicle-treated diabetic mice. n=5 mice in each group.

Supp. Table 4: List of top 40 genes reversed by ATG treatment

Symbol	Log2 Ratio	limma.p	STZ-Vehicle 1	STZ-Vehicle 2	STZ-Vehicle 3	Cont-Vehicle 1	Cont-Vehicle 2	Cont-Vehicle 3	Log2 Rat.io	limma.p.	STZ-ATG 1	STZ-ATG 2	STZ-ATG 3	STZ-Vehicle 1	STZ-Vehicle 2	STZ-Vehicle 3
Ch25h	1.7613	0.0001	9.6598	9.4925	9.0715	7.1995	7.8477	7.7312	-1.2008	0.0237	9.1725	8.2644	8.2918	9.6598	9.4925	9.0715
Abra	1.3099	0.0001	8.6432	8.9183	8.5392	6.8125	7.3824	7.3333	-1.0968	0.0001	7.5464	7.7485	7.5277	8.6432	8.9183	8.5392
Spon2	0.9808	0.0023	9.0717	9.4446	9.4998	8.3263	8.4638	8.7857	-0.9675	0.0021	8.4911	8.4771	7.8121	9.0717	9.4446	9.4998
Dlk1	1.9341	0.0012	8.7705	8.3973	8.6900	6.7559	7.6761	6.6157	-0.9058	0.0093	7.7842	8.2982	7.6213	8.7705	8.3973	8.6900
Adamts9	1.5656	0.0010	11.0678	10.2904	11.1111	9.5022	9.3375	9.6097	-0.8682	0.0296	10.2716	10.1997	10.1327	11.0678	10.2904	11.1111
Sele	1.3699	0.0029	9.3130	8.5979	9.1085	7.0428	8.0370	7.7386	-0.8268	0.0114	8.2817	7.4979	8.3107	9.3130	8.5979	9.1085
Fcer1g	1.0797	0.0053	8.6639	8.5493	8.1898	7.4696	8.0132	7.4000	-0.8119	0.0458	8.3309	7.2349	7.7374	8.6639	8.5493	8.1898
Kctd12b	1.4839	0.0001	12.8755	12.3660	12.8361	11.3366	11.4129	11.3521	-0.8095	0.0076	12.0265	11.9880	12.2463	12.8755	12.3660	12.8361
Laptm5	0.7357	0.0044	9.6010	9.7687	9.2946	8.6473	9.0721	8.8654	-0.7595	0.0233	9.3261	8.5667	8.8415	9.6010	9.7687	9.2946
AW112010	1.0305	0.0012	7.6138	7.6603	7.2095	6.4871	6.8342	6.5833	-0.7259	0.0035	6.9697	6.8879	6.7718	7.6138	7.6603	7.2095
R3hdml	1.4614	0.0000	10.3719	10.1301	10.5718	8.8535	8.9104	9.1154	-0.7225	0.0011	9.7411	9.6134	9.6493	10.3719	10.1301	10.5718
Lhfpl2	1.1563	0.0028	8.5795	8.9214	8.7682	7.6119	7.4192	8.2007	-0.6908	0.0013	8.0066	8.0774	8.2630	8.5795	8.9214	8.7682
Plbd1	0.8100	0.0043	8.9391	8.9054	8.6935	7.6895	8.3777	8.0954	-0.6817	0.0058	8.4018	7.7485	8.2238	8.9391	8.9054	8.6935
Pld4	0.6817	0.0062	8.9988	8.5159	8.2574	7.4249	7.8342	7.8788	-0.6777	0.0387	8.3502	7.7274	7.8383	8.9988	8.5159	8.2574
Cd44	0.8584	0.0046	8.8191	8.6755	8.1997	7.3504	7.8171	7.8586	-0.6599	0.0119	8.0156	7.5980	8.0297	8.8191	8.6755	8.1997
Hdc	2.2571	0.0001	10.3234	9.7365	10.4564	7.6743	8.0663	8.2114	-0.6537	0.0429	9.8087	9.6697	9.5694	10.3234	9.7365	10.4564
Neurl3	1.1377	0.0003	9.6802	9.5677	9.6952	8.5425	8.9486	8.4814	-0.6419	0.0001	9.0660	8.9099	9.0382	9.6802	9.5677	9.6952
Tlr13	1.0811	0.0001	7.7280	7.4904	7.4094	6.6356	6.2961	6.4093	-0.6392	0.0375	7.2411	5.8623	6.8512	7.7280	7.4904	7.4094
Akap12	1.1779	0.0000	12.4497	12.4240	12.6140	11.1888	11.2718	11.4122	-0.6389	0.0013	12.0130	11.5712	11.8108	12.4497	12.4240	12.6140
Ccdc69	0.7615	0.0021	6.8626	6.7846	6.8826	5.7845	6.3731	6.1012	-0.6290	0.0040	6.4868	5.9376	6.2337	6.8626	6.7846	6.8826
Igsf3	-0.7868	0.0026	7.4139	7.6295	7.1699	8.2153	7.9072	8.2007	0.4244	0.0353	7.9255	7.6019	7.8383	7.4139	7.6295	7.1699
Axin2	-0.4091	0.0061	7.2044	6.7705	7.0980	7.5299	7.5072	7.4989	0.4377	0.0219	7.3016	7.9067	7.5357	7.2044	6.7705	7.0980
Dhtkd1	-1.3895	0.0000	6.0624	6.2879	6.0000	7.4519	7.3157	7.6077	0.4409	0.0434	6.2817	6.9739	6.5033	6.0624	6.2879	6.0000
Ppara	-1.2494	0.0005	8.6391	8.9838	8.3332	9.8885	9.7402	10.1859	0.4574	0.0256	9.0528	9.3334	9.0964	8.6391	8.9838	8.3332
Pdgfra	-1.1022	0.0023	9.4898	9.1411	9.9263	10.5994	10.3566	10.5920	0.4729	0.0478	9.9627	10.1907	9.8956	9.4898	9.1411	9.9263
Slc4a8	-0.9066	0.0017	8.7931	9.1749	8.8041	9.4849	9.8612	9.7107	0.4877	0.0201	9.2942	9.2693	9.2918	8.7931	9.1749	8.8041
Map3k13	-0.9316	0.0008	6.7590	6.5410	6.4919	7.1781	7.4726	7.6157	0.5133	0.0409	7.0543	7.1530	6.6877	6.7590	6.5410	6.4919
Tns1	-0.8735	0.0005	14.3248	14.6981	14.3341	15.2076	15.1793	15.2980	0.5133	0.0126	14.8786	14.8474	14.8269	14.3248	14.6981	14.3341
Mgat3	-1.0233	0.0007	8.5664	8.6830	8.7448	9.7064	9.4169	10.0911	0.5241	0.0227	9.2072	9.4081	8.8054	8.5664	8.6830	8.7448
Nkg7	-1.2077	0.0009	6.9593	7.2170	6.6147	8.3835	8.1670	7.8104	0.5275	0.0287	7.4868	7.2249	7.4868	6.9593	7.2170	6.6147
Rasgef1a	-1.0409	0.0004	6.8118	6.8260	7.1799	7.8669	7.9265	7.6823	0.5398	0.0354	7.1118	7.4938	7.3658	6.8118	6.8260	7.1799
Angptl7	-1.0930	0.0004	7.1702	6.7846	6.8826	8.4608	7.9392	7.9756	0.5791	0.0105	7.5547	7.2448	7.4617	7.1702	6.7846	6.8826
Slc16a14	-1.9529	0.0006	5.7590	5.8794	5.4594	7.7119	7.1286	8.4503	0.6382	0.0404	6.3972	6.8879	5.8512	5.7590	5.8794	5.4594
Rab11fip4	-1.4835	0.0005	8.0624	8.6565	7.8642	9.5918	9.4704	9.5460	0.6472	0.0438	8.5589	8.7096	8.9864	8.0624	8.6565	7.8642
Coro2a	-0.3645	0.0032	10.1824	10.4053	10.4671	10.7698	10.7474	10.8282	0.6479	0.0005	11.0532	11.1762	10.9349	10.1824	10.4053	10.4671
Prox1	-1.6093	0.0000	6.4898	6.5738	6.4919	8.1288	7.6609	8.1012	0.6506	0.0001	7.1005	7.1425	7.1731	6.4898	6.5738	6.4919
Acmsd	-2.2121	0.0015	7.4332	7.8729	6.7682	9.6454	8.7681	9.9756	0.6672	0.0448	8.1005	8.8132	7.8054	7.4332	7.8729	6.7682
Ptpru	-0.6496	0.0014	10.9391	11.3564	11.1134	11.7263	11.7814	11.7630	0.7006	0.0198	11.8393	11.8141	11.3439	10.9391	11.3564	11.1134
Sypl2	-1.9381	0.0001	6.4712	6.7419	5.9069	8.5508	8.0370	8.4093	0.7698	0.0276	7.2411	7.4213	6.7163	6.4712	6.7419	5.9069
Trpm6	-1.5934	0.0034	10.1702	10.8546	9.7330	11.8922	11.7636	11.2907	0.7864	0.0406	10.8009	10.9566	11.1267	10.1702	10.8546	9.7330

Supp. Table 5: Top 20 GO biological processes of differentially expressed genes (DEGs) in glomeruli of diabetic mice that are reversed by ATG treatment

Term	P-value	Adjusted P-value	Z-score
leukocyte migration (GO:0050900)	2.18E-08	5.04E-05	-2.2705
leukocyte cell-cell adhesion (GO:0007159)	5.61E-06	6.50E-03	-2.2177
regulation of cell activation (GO:0050865)	6.56E-05	1.27E-02	-2.5017
neutrophil activation (GO:0042119)	2.11E-04	1.99E-02	-2.7702
positive regulation of cytokine production (GO:0001819)	4.69E-05	1.21E-02	-2.4340
leukocyte activation (GO:0045321)	1.56E-05	1.08E-02	-2.3745
regulation of mast cell degranulation (GO:0043304)	1.76E-04	1.94E-02	-2.6926
actin filament-based process (GO:0030029)	2.02E-05	1.08E-02	-2.3232
granulocyte activation (GO:0036230)	2.95E-04	1.99E-02	-2.6685
regulation of mast cell activation involved in immune response (GO:0033006)	2.11E-04	1.99E-02	-2.6196
integrin-mediated signaling pathway (GO:0007229)	2.79E-05	1.08E-02	-2.2395
myeloid leukocyte activation (GO:0002274)	3.26E-05	1.08E-02	-2.2333
single organism cell adhesion (GO:0098602)	6.39E-05	1.27E-02	-2.3149
blood coagulation (GO:0007596)	8.31E-05	1.38E-02	-2.3514
coagulation (GO:0050817)	8.31E-05	1.38E-02	-2.3488
cell-substrate adhesion (GO:0031589)	2.89E-05	1.08E-02	-2.2066
neutrophil chemotaxis (GO:0030593)	4.68E-05	1.21E-02	-2.2405
hemostasis (GO:0007599)	9.69E-05	1.50E-02	-2.3452
neutrophil migration (GO:1990266)	5.25E-05	1.22E-02	-2.2101
regulation of leukocyte activation (GO:0002694)	3.02E-04	1.99E-02	-2.4414

Supp. Table 6: Top 20 Wikipathways (2015) of differentially expressed genes (DEGs) in glomeruli of diabetic mice that are reversed by ATG treatment

Term	P-value	Adjusted P-value	Z-score
Adipogenesis genes(Mus musculus)	0.0046	0.3318	-1.9659
IL-3 Signaling Pathway(Homo sapiens)	0.0085	0.3318	-1.9585
Integrin-mediated Cell Adhesion(Homo sapiens)	0.0054	0.3318	-1.9371
Integrin-mediated Cell Adhesion(Mus musculus)	0.0047	0.3318	-1.9286
Wnt Signaling Pathway Netpath(Homo sapiens)	0.0096	0.3318	-1.8352
Arrhythmogenic Right Ventricular Cardiomyopathy(Homo sapiens)	0.0069	0.3318	-1.7652
Adipogenesis(Homo sapiens)	0.0198	0.4542	-1.7763
Focal Adhesion(Homo sapiens)	0.0276	0.5160	-1.8580
Focal Adhesion(Mus musculus)	0.0256	0.5160	-1.8566
Integrated Breast Cancer Pathway(Homo sapiens)	0.0324	0.5160	-1.7476
MAPK signaling pathway(Mus musculus)	0.0360	0.5324	-1.8343
TWEAK Signaling Pathway(Homo sapiens)	0.0309	0.5160	-1.6900
MAPK Signaling Pathway(Homo sapiens)	0.0554	0.5871	-1.8082
Nucleotide GPCRs(Mus musculus)	0.0162	0.4180	-0.9617
Regulation of toll-like receptor signaling pathway(Homo sapiens)	0.0806	0.5871	-1.5145
RANKL/RANK Signaling Pathway(Homo sapiens)	0.0559	0.5871	-1.4573
Wnt Signaling Pathway and Pluripotency(Mus musculus)	0.0605	0.5871	-1.4273
Toll-like receptor signaling pathway(Homo sapiens)	0.0827	0.5871	-1.4124
Neural Crest Differentiation(Homo sapiens)	0.0763	0.5871	-1.3840
Nucleotide GPCRs(Homo sapiens)	0.0162	0.4180	-0.7966

Supp. Table 7: Top 20 KEGG pathways (2015) differentially expressed genes (DEGs) in glomeruli of diabetic mice that are reversed by ATG treatment

Term	P-value	Adjusted P-value	Z-score
natural killer cell mediated cytotoxicity	0.000376	0.015133	-1.99412
cell adhesion molecules	0.000466	0.015133	-1.82481
cytokine cytokine receptor interaction	0.002954	0.064008	-1.95691
colorectal cancer	0.015181	0.246684	-1.83668
mapk signaling pathway	0.024233	0.31503	-1.91543
pancreatic cancer	0.037633	0.330152	-1.62302
adipocytokine signaling pathway	0.034633	0.330152	-1.5672
jak stat signaling pathway	0.040634	0.330152	-1.53657
apoptosis	0.051095	0.369017	-1.39187
small cell lung cancer	0.058701	0.381558	-1.36026
hematopoietic cell lineage	0.064785	0.38282	-1.20971
toll like receptor signaling pathway	0.089795	0.426244	-1.22122
glioma	0.091806	0.426244	-1.08802
b cell receptor signaling pathway	0.091806	0.426244	-0.98996
regulation of actin cytoskeleton	0.125741	0.527457	-1.2084
chronic myeloid leukemia	0.13795	0.527457	-0.99764
calcium signaling pathway	0.159513	0.53653	-0.91293
adherens junction	0.13795	0.527457	-0.8516
prostate cancer	0.181979	0.53653	-0.78271
focal adhesion	0.225886	0.53653	-0.62749

Supp. Table 8: Proteins identified from DARTS and mass spectrometry

Identified Proteins (3004)	Accession ID	Molecular Weight	Spectra counts (ATG 10μM)	Spectra counts (DMSO)	Ratio (ATG/ DMSO)
Tubulin beta-3 chain OS=Homo sapiens GN=TUBB3 PE=1 SV=2	TBB3_HUMAN	50 kDa	49	0	25.6
Tubulin beta-6 chain OS=Homo sapiens GN=TUBB6 PE=1 SV=1	TBB6_HUMAN	50 kDa	25	0	13.6
**Serine/threonine-protein phosphatase 2A catalytic subunit beta isoform OS=Homo sapiens GN=PPP2CB PE=1 SV=1**	**PP2AB_HUMAN**	**36 kDa**	**14**	**0**	**7.8**
Heterogeneous nuclear ribonucleoprotein H2 OS=Homo sapiens GN=HNRNPH2 PE=1 SV=1	HNRH2_HUMAN	49 kDa	12	0	6.8
Keratin, type II cytoskeletal 6A OS=Homo sapiens GN=KRT6A PE=1 SV=3	K2C6A_HUMAN	60 kDa	8	0	5.2
Keratin, type I cytoskeletal 17 OS=Homo sapiens GN=KRT17 PE=1 SV=2	K1C17_HUMAN	48 kDa	7	0	4.7
Protein transport protein Sec61 subunit alpha isoform 1 OS=Homo sapiens GN=SEC61A1 PE=1 SV=2	S61A1_HUMAN	52 kDa	7	0	4.7
Keratin, type II cytoskeletal 5 OS=Homo sapiens GN=KRT5 PE=1 SV=3	K2C5_HUMAN	62 kDa	7	0	4.7

Supp. Table 9: PP2A interacting proteins by IP/mass spectrometry

	Identified Proteins (top 20 of total 1033)	Accession Number	MW	Spectra counts (EV)	Spectra counts (PP2A)	Ratio (PP2A/EV)
**1**	**Serine/threonine-protein phosphatase 2A 65 kDa regulatory subunit A alpha isoform OS=Homo sapiens GN=PPP2R1A PE=1 SV=4**	P30153	65 kDa	5	406	58.3
2	**Tubulin beta-4A chain OS=Homo sapiens GN=TUBB4A PE=1 SV=2**	P04350	50 kDa	0	62	32
**3**	**Serine/threonine-protein phosphatase 2A 65 kDa regulatory subunit A beta isoform OS=Homo sapiens GN=PPP2R1B PE=1 SV=3**	P30154	66 kDa	0	48	25
4	Ferrochelatase, mitochondrial OS=Homo sapiens GN=FECH PE=1 SV=2	P22830	48 kDa	0	11	6.5
**5**	**CTP synthase 1 OS=Homo sapiens GN=CTPS1 PE=1 SV=2**	P17812	67 kDa	0	11	6.5
**6**	**Serine/threonine-protein phosphatase 2A 56 kDa regulatory subunit delta isoform OS=Homo sapiens GN=PPP2R5D PE=1 SV=1**	Q14738	70 kDa	0	10	6
**7**	**Guanine nucleotide-binding protein G(k) subunit alpha OS=Homo sapiens GN=GNAI3 PE=1 SV=3**	P08754	41 kDa	0	10	6
8	**Neutral alpha-glucosidase AB OS=Homo sapiens GN=GANAB PE=1 SV=3**	Q14697	107 kDa	0	9	5.5
9	Eukaryotic initiation factor 4A-II OS=Homo sapiens GN=EIF4A2 PE=1 SV=2	Q14240	46 kDa	0	8	5
**10**	**Interferon-induced GTP-binding protein Mx1 OS=Homo sapiens GN=MX1 PE=1 SV=4**	P20591	76 kDa	0	7	4.5
11	Histone H2A type 1-B/E OS=Homo sapiens GN=HIST1H2AB PE=1 SV=2	P04908 (+2)	14 kDa	0	7	4.5
12	HLA class I histocompatibility antigen, B-41 alpha chain OS=Homo sapiens GN=HLA-B PE=1 SV=1	P30479	41 kDa	0	6	4
**13**	**Gamma-interferon-inducible protein 16 OS=Homo sapiens GN=IFI16 PE=1 SV=3**	Q16666	88 kDa	0	5	3.5
14	60S ribosomal protein L23 OS=Homo sapiens GN=RPL23 PE=1 SV=1	P62829	15 kDa	0	5	3.5
15	14-3-3 protein gamma OS=Homo sapiens GN=YWHAG PE=1 SV=2	P61981	28 kDa	0	5	3.5
**16**	**Cytoskeleton-associated protein 4 OS=Homo sapiens GN=CKAP4 PE=1 SV=2**	Q07065	66 kDa	1	8	3.3
**17**	**Drebrin OS=Homo sapiens GN=DBN1 PE=1 SV=4**	Q16643	71 kDa	2	10	3
18	HLA class I histocompatibility antigen, B-44 alpha chain OS=Homo sapiens GN=HLA-B PE=1 SV=1	P30481	40 kDa	1	7	3
19	Poly [ADP-ribose] polymerase 9 OS=Homo sapiens GN=PARP9 PE=1 SV=2	Q8IXQ6	96 kDa	0	4	3
**20**	**Interferon-induced protein with tetratricopeptide repeats 3 OS=Homo sapiens GN=IFIT3 PE=1 SV=1**	O14879	56 kDa	0	4	3

Supp. Table 10: Quantification estimates (%) of phospho-peptides in Dbn1 by mass spectrometry

Putative phosphorylation site: **S142** (LS**S**PVLHR)		
	**Percentage (%)**	**PhosphoRS: Best site probabilities (%)**
Vehicle	94.0	98.45
ATG	75.0	100.0
PP2A KD	100.0	100.0

Putative phosphorylation site: **T335** or **S337** (MAPTPIP**T**R**S**PDSSTASTPVAEQIER)		
	**Percentage (%)**	**PhosphoRS: Best site probabilities (%)**
Vehicle	25.8	*49.38 (T335), 49.38 (S337)
ATG	13.0	*49.50 (T335), 49.50 (S337)
PP2A KD	36.5	*50 (T335), 50 (S337)

*The analysis confirmed that the peptide was singly phosphorylated (FDR<0.01) but could not distinguish whether T335 or S337 was the site of phosphorylation.

Supp. Table 11: Blood glucose and kidney-to-body weight ratio

Mouse groups	Blood glucose (mg/dl)	KW/BW (mg/g)
WT (-STZ)	119.4±35.6	6.65±0.9
Pod-PP2A$^{-/-}$(-STZ)	132±23.3	6.87±0.65
WT (+STZ)	562.7±74.5***	9.38±1.52*
Pod-PP2A$^{-/-}$(+STZ)	564±67.4***	11.26±1.42**

*P<0.05, *P<0.01, and ***P<0.001 when compared to non-diabetic mice. n=6, in each group.

Supp. Table 12: Primers used for real-time PCR

Primer	Sequence
mFgr-F	5’-ACAGAGGCTATGTTCCCAGCA-3’
mFgr-R	5’-GGGGTTACCAGAGGACAGAAG-3’
mItgb7-F	5’-AGGACGACTTGGAACGTGTG-3’
mItgb7-R	5’-ATGGGTGGTGAAGCTTGGAG-3’
mZyx-F	5’-TCCTCACTGCTGGACGACAT-3’
mZyx-R	5’-GGGCAGGTTTGGTACTAGGC-3’
mWas-F	5’-CACCTGTGCCCCCACAC-3’
mWas-R	5’-CGGTGCTCCGATATCAGCTT-3’
mWash1-F	5’-ACAGGATCCAGAGCAAGCAC-3’
mWash1-R	5’-TGTTGAAGAGCAGCAGGGAG-3’
