# Supplementary material for: Arctigenin attenuates diabetic kidney disease through the activation of PP2A in podocytes
Source: Nat Commun. 2019 Oct 4;10:4523. doi: 10.1038/s41467-019-12433-w (PMC6778111; doi:10.1038/s41467-019-12433-w)
Supplement: Supplementary file 2 — Reporting Summary [file 41467_2019_12433_MOESM2_ESM.pdf]

nature research

Corresponding author(s): John C. He

Last updated by author(s): July 16, 2019

# Reporting Summary

Nature Research wishes to improve the reproducibility of the work that we publish. This form provides structure for consistency and transparency in reporting. For further information on Nature Research policies, see Authors & Referees and the Editorial Policy Checklist.

## Statistics

For all statistical analyses, confirm that the following items are present in the figure legend, table legend, main text, or Methods section.

| n/a | Confirmed | |
|---|---|---|
| ☐ | ☒ | The exact sample size (*n*) for each experimental group/condition, given as a discrete number and unit of measurement |
| ☐ | ☒ | A statement on whether measurements were taken from distinct samples or whether the same sample was measured repeatedly |
| ☐ | ☒ | The statistical test(s) used AND whether they are one- or two-sided<br>*Only common tests should be described solely by name; describe more complex techniques in the Methods section.* |
| ☒ | ☐ | A description of all covariates tested |
| ☐ | ☒ | A description of any assumptions or corrections, such as tests of normality and adjustment for multiple comparisons |
| ☐ | ☒ | A full description of the statistical parameters including central tendency (e.g. means) or other basic estimates (e.g. regression coefficient) AND variation (e.g. standard deviation) or associated estimates of uncertainty (e.g. confidence intervals) |
| ☒ | ☐ | For null hypothesis testing, the test statistic (e.g. $F$, $t$, $r$) with confidence intervals, effect sizes, degrees of freedom and $P$ value noted<br>*Give P values as exact values whenever suitable.* |
| ☒ | ☐ | For Bayesian analysis, information on the choice of priors and Markov chain Monte Carlo settings |
| ☒ | ☐ | For hierarchical and complex designs, identification of the appropriate level for tests and full reporting of outcomes |
| ☒ | ☐ | Estimates of effect sizes (e.g. Cohen's *d*, Pearson's *r*), indicating how they were calculated |

*Our web collection on statistics for biologists contains articles on many of the points above.*

## Software and code

Policy information about availability of computer code

| | |
|---|---|
| Data collection | PubChem (https://pubchem.ncbi.nlm.nih.gov/) |
| Data analysis | ChemBioDraw software (PerkinElmer) |

For manuscripts utilizing custom algorithms or software that are central to the research but not yet described in published literature, software must be made available to editors/reviewers. We strongly encourage code deposition in a community repository (e.g. GitHub). See the Nature Research guidelines for submitting code & software for further information.

## Data

Policy information about availability of data

All manuscripts must include a data availability statement. This statement should provide the following information, where applicable:

- Accession codes, unique identifiers, or web links for publicly available datasets
- A list of figures that have associated raw data
- A description of any restrictions on data availability

RNA-seq data that support the findings of this study have been deposited in the Gene Expression Omnibus (GEO) repository under the accession # GSE134327 (https://www.ncbi.nlm.nih.gov/geo/query/acc.cgi?acc=GSE134327). The mass spectrometry proteomic dataset is deposited to the ProteomeXchange Consortium via the Proteomics IDEntifications (PRIDE)partner repository with the dataset identifier PXD014976 and 10.6019/PXD014976. All other data generated or analyzed during this study are included in this published article (and its supplementary information files). The source data underlying Figs 1b, 1d, 2b, 2d, 3a, 3c-f, 4a-d, 5a-f, 6b, 6d, 7b, 7d, 8b, 8e, and Supplementary Figs 1, 2, 4, and 7 are provided as a Source Data file. All data are also available from the corresponding author upon reasonable request.

# Field-specific reporting

Please select the one below that is the best fit for your research. If you are not sure, read the appropriate sections before making your selection.

☒ Life sciences ☐ Behavioural & social sciences ☐ Ecological, evolutionary & environmental sciences

For a reference copy of the document with all sections, see nature.com/documents/nr-reporting-summary-flat.pdf

# Life sciences study design

All studies must disclose on these points even when the disclosure is negative.

| | |
|---|---|
| Sample size | Sample sizes were chosen based on previous mouse experiments to detect sufficient differences in the renal outcome. |
| Data exclusions | n/a |
| Replication | Additional batch of experiments were performed on mice, which gave reproducible results. |
| Randomization | Mice were randomly selected for treatments (drug or vehicle). |
| Blinding | Investigators were blinded to the analysis of sample groups. |

# Reporting for specific materials, systems and methods

We require information from authors about some types of materials, experimental systems and methods used in many studies. Here, indicate whether each material, system or method listed is relevant to your study. If you are not sure if a list item applies to your research, read the appropriate section before selecting a response.

## Materials & experimental systems

| n/a | Involved in the study | |
|---|---|---|
| ☐ | ☒ | Antibodies |
| ☐ | ☒ | Eukaryotic cell lines |
| ☒ | ☐ | Palaeontology |
| ☐ | ☒ | Animals and other organisms |
| ☒ | ☐ | Human research participants |
| ☒ | ☐ | Clinical data |

## Methods

| n/a | Involved in the study | |
|---|---|---|
| ☒ | ☐ | ChIP-seq |
| ☒ | ☐ | Flow cytometry |
| ☒ | ☐ | MRI-based neuroimaging |

## Antibodies

| | |
|---|---|
| Antibodies used | Antibodies against: WT-1 (Santa Cruz Sc-192), collagen I (Millipore: ab756p), phospho-p65 (Abcam, ab28856), total p65 (Cell Signaling, 4764), PP2A (Abcam, ab168350), GAPDH (Cell Signaling, 2118), Drebrin-1 (Cell signaling, 5202) and beta-actin (Sigma, A5136) |
| Validation | All of the antibodies used were validated either directly by the commercial manufacturers or by cited references included on the antibody information for the specified uses in our manuscript. |

## Eukaryotic cell lines

Policy information about cell lines

| | |
|---|---|
| Cell line source(s) | Immortalized human podocytes were obtained from Dr. Moin Saleem (UK). |
| Authentication | Cell lines were not authenticated by us, as they were previously characterized. |
| Mycoplasma contamination | Mycoplasma testing is conducted every 4 month intervals |
| Commonly misidentified lines (See ICLAC register) | *Name any commonly misidentified cell lines used in the study and provide a rationale for their use.* |

## Animals and other organisms

Policy information about studies involving animals; ARRIVE guidelines recommended for reporting animal research

| | |
|---|---|
| Laboratory animals | Male eNOS homozygous knockout (eNOS-/-) mice on a C57BL/6J background and db/db mice in BLKS background were purchased from The Jackson Laboratory. Diabetes were induced in mice at 8 weeks of age. Inducible podocyte-specific PP2A knockout mice were generated by crossing PP2A floxed mice (PP2A regulatory subunit A alpha;Ppp2r1a) with NPHS2-rtTA and tetO-Cre transgenic mice; all three mouse lines were in the FVB/NJ background and obtained from The Jackson Laboratory. Diabetes was induced in mice at 8 weeks of age. |
| Wild animals | n/a |
| Field-collected samples | *For laboratory work with field-collected samples, describe all relevant parameters such as housing, maintenance, temperature, photoperiod and end-of-experiment protocol OR state that the study did not involve samples collected from the field.* |
| Ethics oversight | All procedures performed in the study involving animals were in accordance with the ethical standards of and protocol (#15-1402) approved by the Institutional Animal Care and Use Committee at the Icahn School of Medicine at Mount Sinai, New York, NY. |

Note that full information on the approval of the study protocol must also be provided in the manuscript.
